# Methacrylated gelatin and platelet-rich plasma based hydrogels promote regeneration of critical-sized bone defects

**DOI:** 10.1093/rb/rbae022

**Published:** 2024-03-05

**Authors:** Shichao Lian, Zhiyu Mu, Zhengchao Yuan, Muhammad Shafiq, Xiumei Mo, Weidong Mu

**Affiliations:** Department of Traumatic Orthopaedics, Shandong Provincial Hospital, Shandong University, Jinan, Shandong 250012, China; Zoucheng People’s Hospital, Zoucheng, Shandong 273500, China; Department of Medical Physics and Biomedical Engineering, University of London, London WC1E 6BT, UK; State Key Laboratory for Modification of Chemical Fibers and Polymer Materials, Shanghai Engineering Research Center of Nano-Biomaterials and Regenerative Medicine, College of Biological Science and Medical Engineering, Donghua University, Shanghai 201620, PR China; Innovation Center of NanoMedicine (iCONM), Kawasaki Institute of Industrial Promotion, Kawasaki-Ku, Kawasaki 210-0821, Japan; State Key Laboratory for Modification of Chemical Fibers and Polymer Materials, Shanghai Engineering Research Center of Nano-Biomaterials and Regenerative Medicine, College of Biological Science and Medical Engineering, Donghua University, Shanghai 201620, PR China; Department of Traumatic Orthopaedics, Shandong Provincial Hospital, Shandong University, Jinan, Shandong 250012, China

**Keywords:** large-sized bone defect repair, platelet-rich plasma, hydrogel, tissue scaffold, gelatin methacrylate

## Abstract

Physiological repair of large-sized bone defects requires instructive scaffolds with appropriate mechanical properties, biocompatibility, biodegradability, vasculogenic ability and osteo-inductivity. The objective of this study was to fabricate *in situ* injectable hydrogels using platelet-rich plasma (PRP)-loaded gelatin methacrylate (GM) and employ them for the regeneration of large-sized bone defects. We performed various biological assays as well as assessed the mechanical properties of GM@PRP hydrogels alongside evaluating the release kinetics of growth factors (GFs) from hydrogels. The GM@PRP hydrogels manifested sufficient mechanical properties to support the filling of the tissue defects. For biofunction assay, the GM@PRP hydrogels significantly improved cell migration and angiogenesis. Especially, transcriptome RNA sequencing of human umbilical vein endothelial cells and bone marrow-derived stem cells were performed to delineate vascularization and biomineralization abilities of GM@PRP hydrogels. The GM@PRP hydrogels were subcutaneously implanted in rats for up to 4 weeks for preliminary biocompatibility followed by their transplantation into a tibial defect model for up to 8 weeks in rats. Tibial defects treated with GM@PRP hydrogels manifested significant bone regeneration as well as angiogenesis, biomineralization, and collagen deposition. Based on the biocompatibility and biological function of GM@PRP hydrogels, a new strategy is provided for the regenerative repair of large-size bone defects.

## Graphical abstract

**Figure:**
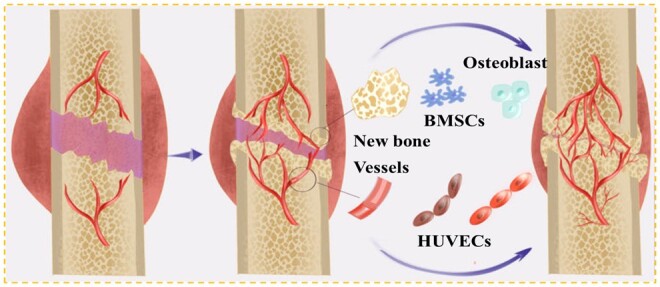

## Introduction

Bone, as a mineralized connective tissue with self-healing properties displays complex physiological functions, such as protections of the organs and soft tissues and support for the movement of the body [1]. Large-sized musculoskeletal defects (>3 cm) remain a perpetual challenge which adversely impact the quality of life [2]. Especially, large-sized bone defects induced by the traumatic injuries, osteoporosis, or surgical removal of bone tumors may exhibit poor reparability plausibly due to the poor host integration, and fibrocartilage formation [3, 4]. Generally, autografts, allografts and non-biological materials are utilized for the reconstruction of large bone defects [5]. Bone, as a mineralized connective tissue with self-healing properties displays complex physiological functions. In the USA alone, approximately 200.000 autografts are annually used albeit several complications during grafting procedures [1]. While appealing, each of these categories of reconstructive materials has its own limitations: (i) Autografts possess osteogenic, osteo-inductive, and osteoconductive abilities, while lack of appropriate transplantable allografts as well as immunological risks impede their full translation. (ii) non-biological materials, such as bone cement could manage infected or uninfected bone defects albeit their poor abilities for bone integration and slow remodeling [5, 6]. In contrast, bioactive materials with good biocompatibility and biodegradability, stability, and concurrent osteogenesis and vasculogenic abilities hold great promise for the regeneration of large-sized bone defects [6, 7]. An ideal scaffold for bone repair should exhibit certain characteristics including: (i) sufficient mechanical integrity to confer a stable cell regenerative microenvironment, (ii) osteo-conductivity and osteo-inductivity to promote biomineralization, (iii) biodegradability, and (iv) biocompatibility [8, 9].

Hydrogels exhibit large water content as well as mechanical properties similar to the soft biological tissues. Consequently, hydrogels have been widely exploited for the regeneration of injured soft and hard tissue, including osteochondral and musculoskeletal defects [10, 11]. Especially, natural and synthetic polymers have been extensively utilized to develop scaffolds for bone tissue repair. Methacrylated gelatin (GM) has received considerable attention of the research community owing to its biocompatibility, biodegradability, and mechanically tunability [12, 13]. The GM can be further processed into hydrogels and three dimensional (3D) scaffolds through photopolymerization using appropriate photoinitiators [14–16]. The GM-based hydrogels have also been exploited for the spatio-temporal release of bioactive cues, such as growth factors (GFs), exosomes, and antibiotic therapeutics [14, 17]. Despite these advantages, the GM hydrogels show limited cell infiltration after transplantation and lack effective vascularization to support osteogenesis process *in vivo*, which necessitates the advent of alternative strategies, especially, the incorporation of bioactive cues [18, 19].

Recently, there has been a surge in the use of platelet-rich plasma (PRP) for regenerative medicine and tissue engineering (TE) applications partly owing to its ability to release different types of GFs; the latter may play a significant role to promote cell migration, proliferation, and differentiation [20, 21]. The PRP is an autologous yet an economical biological material, which contains diverse cytokines, including platelet-derived growth factor (PDGF), vascular endothelial growth factor (VEGF), transforming growth factor-beta1 (TGF-β1), and insulin-like growth factor-1 [20, 22]. These GFs may actively participate in tissue repair by recruiting and activating different types of cells alongside promoting the formation of angiogenic networks. Intriguingly, platelets may also impart antimicrobial and immune-modulation characteristics to the scaffolds, which may further suppress infection and facilitate tissue repair [23, 24]. The PRP has also been shown to promote the migration, proliferation, and differentiation of human umbilical vein endothelial cells (HUVECs) and bone marrow-derived mesenchymal stem cells (BMSC); the latter may also be beneficial for bone repair owing to their abilities to differentiate into bone forming cells as well as induce biomineralization [25, 26]. Nonetheless, PRP lacks an appropriate application form for a sustained therapeutic effect. Therefore, PRP needs to be effectively delivered along with the biomaterials to facilitate osteochondral tissue repair.

**Figure 1.**
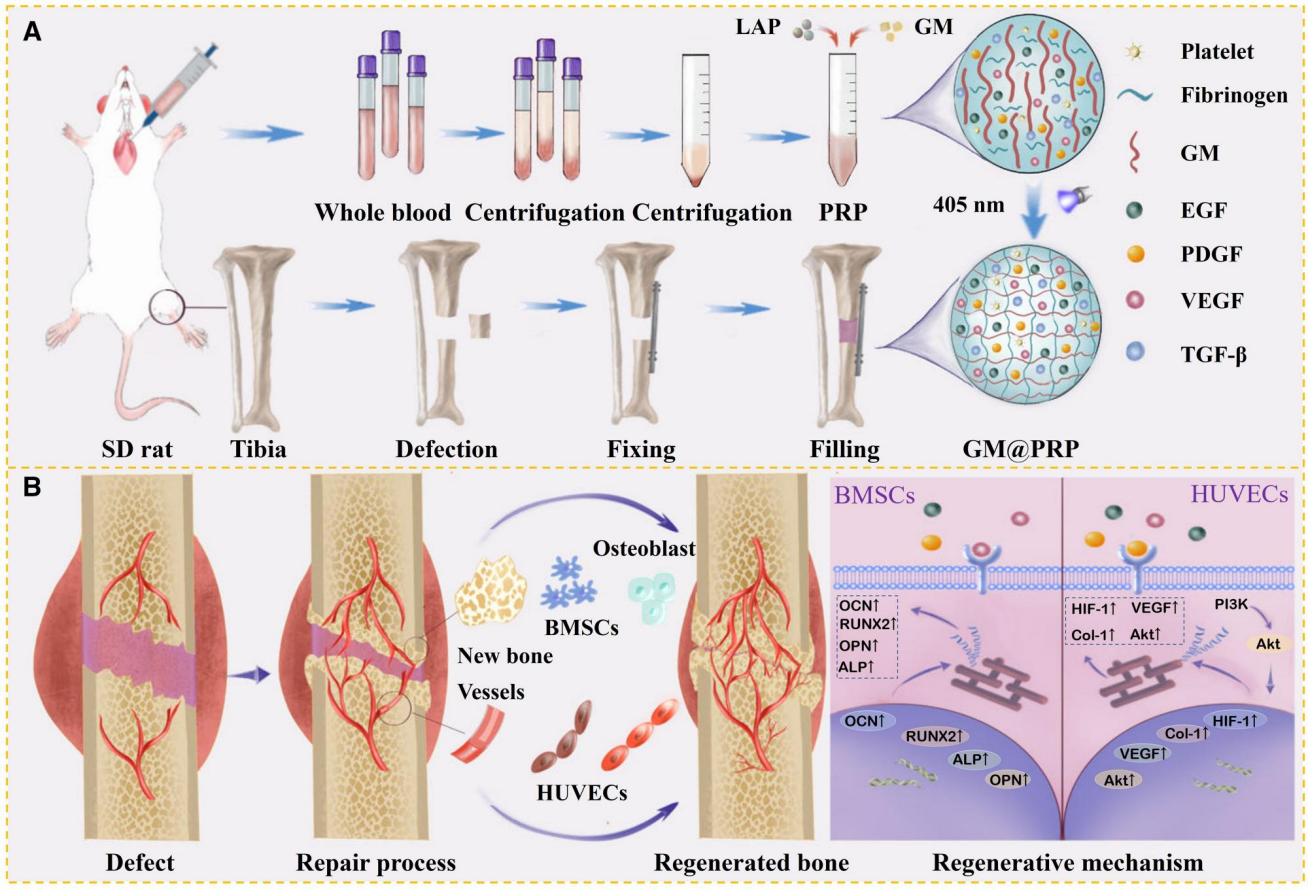
An overview of the experimental design. **(A)** Isolation of PRP from whole blood and preparation of a segmental bone defect model. **(B)** An illustration showing dynamic and interactive healing process of GM@PRP hydrogels during bone repair.

The objective of this research was to fabricate *in situ* injectable hydrogels using PRP-loaded GM and employ them for the regeneration of a large-sized bone defect. We performed a series of biological assays as well as assessed the mechanical properties of GM@PRP hydrogels alongside evaluating the release kinetics of GFs from hydrogels. The GM@PRP hydrogels exhibited sufficent mechanical properties to support the filling of tissue defects along with the release of various types of GFs. The GM@PRP hydrogels also improved cell migration and angiogenesis *invitro*. Transcriptome RNA sequencing of HUVECs and BMSCs was performed to delineate the vascularization and biomineralization abilities of GM@PRP hydrogels. The GM@PRP hydrogels were subcutaneously implanted in rats for up to 4 weeks for preliminary biocompatibility assessment followed by their transplantation into a tibial defect model for up to 8 weeks in rats (Figure 1B). Tibial defects treated with GM@PRP hydrogels manifested significant bone regeneration as well as angiogenesis, biomineralization, and collagen deposition. Based on the biocompatibility and biological function of GM@PRP hydrogels, a new strategy is provided for the regenerative repair of large-size bone defects. These GM@PRP hydrogels may offer an invaluable platform to simultaneously promote vascularization and osteogenesis, which may also have implications for the regeneration large-sized bone defects.

## Experimental

### Materials

Lithium phenyl-2,4,6-trimethylbenzoylphosphinate (LAP) and GelMA (GM, EFL-GM-90, DS: 90%) were obtained from Yongqinquan Intelligent Equipment Co., Ltd (Suzhou, China). HUVECs were purchased from the Typical Culture Collection Committee Cell Bank, Chinese Academy of Sciences (Shanghai, China). The rBMSCs were isolated from Sprague–Dawley (SD) rats following our previous report [27]. The fetal bovine serum (FBS, BC-SE-FBS01) was purchased from BioChannel Biotechnology (Nanjing, China). Dulbecco’s modified eagle medium (DMEM) high-sugar complete medium was obtained from Beijing Solarbio Science & Technology Co., Ltd (Beijing, China). Alkaline phosphatase (ALP) assay kit (P0321), BCA protein assay kit (P0012S), 5-bromo-4-chloro-3-indolyl phosphate (BCIP)/nitro blue tetrazolium (NBT) analysis kit (C3206), and Alizarin red S staining kit (ARS, R20796) were purchased from Beyotime Biotechnology (Shanghai, China). Enzyme-linked immunosorbent assay (ELISA) kits for epidermal GF (EGF), PDGF, and TGF-β1 were obtained from Jiangsu Baolai Biotechnology Co., Ltd (catalog no. MM-02 3902, 02 9802, and 02 4802, Jiangsu, China). All other reagents were of analytical grade and used without further purification.

### Preparation of PRP and GM@PRP hydrogels

To prepare PRP, a total 10 ml of blood was collected from the carotid artery of SD rats and supplemented with sodium citrate before centrifugation at 200 *g* for 10 min under sterile conditions. The supernatant containing a thin layer of erythrocytes and platelets was collected and transferred to another tube. The platelets were further enriched with an additional centrifugation cycle at 600 *g* for 8 min. Approximately one-third (1/3) portion of the supernatant was remained after the removal of erythrocytes (Supplementary Figure S1).

To optimize the ratio of PRP and GM, different types of hydrogels were prepared (Supplementary Table S1). The GM@PRP hydrogels (GM: PRP = 1:1, *v/v*) displayed an appropriate pore size and gelatinization strength, which were used for the subsequent experiments. Briefly, GM was resuspended in phosphate-buffered saline (PBS) to afford a final concentration of 20% (*w/v*) and was incubated in a water bath at 50°C until the complete dissolution. The LAP (0.05%, *w/v*) was employed for the crosslinking of the GM The GM/LAP solution was heated at 37°C in a water bath and sterilized with a 0.22 μm filter paper and then stored at 4°C. For the preparation of GM@PRP hydrogels, GM was blended along with LAP and PRP solutions (GM: PRP = 1:1, *v/v*) to obtain a homogenous mixture, and stored at 4°C for the subsequent use. The GM and GM@PRP hydrogels were poured into the mold and cross-linked at 405 nm to realize various cylindrical hydrogels.

### Physicochemical characterization of hydrogels

#### Morphology of GM and GM@PRP hydrogels

The surface morphology of GM and GM@PRP hydrogels was evaluated by scanning electron microscopy (SEM, Hitachi TM-1000, Tokyo, Japan). The GM and GM@PRP hydrogels were lyophilized using a freeze-drier (SCIENTZ-10N, Scientz, Ningbo, China) and gold-sputtered before microscopic analysis. For degradation assay, cylindrical-shaped hydrogels were prepared by gelating a total 200 µl of GM or GM@PRP hydrogel precursor solutions. Samples were immersed into a total 5 ml of PBS and incubated in an automatic shaker at 37°C. At pre-determined time points, the samples were freeze-dried for SEM analysis. SEM images were further analyzed by ImageJ (ImageJ 1.51) software to calculate the pore size.

#### Release kinetics and degradation of GM@PRP hydrogels

About 100 µl of GM@PRP hydrogel precursor solution (*n *=* *5) was transferred into an Eppendorf tube (EP) tube. After gelation, 1.2 ml of PBS was added into the tube and stirred in an incubator at 37°C and 120 rotations per minute (rpm). At pre-determined time points, the supernatant was collected and an equal volume of PBS was added to maintain an infinite sink condition. The release of EGF, PDGF, and TGF-β1 from GM@PRP hydrogels was assessed by corresponding ELISA kits following manufacturer’s instructions. Moreover, the standard curves of the above-mentioned GFs were prepared as shown in Supplementary Figure S2.

The degradation of the hydrogels was evaluated for up to 35 days *in* vitro. Approximately, 500 μl of GM or GM@PRP hydrogel precursor solutions were poured into the mold and crosslinked to obtain the hydrogel under sterile conditions. The initial weight of the GM or GM@PRP hydrogels was measured and recorded as *A*_0_, Samples were then soaked in 5 ml of PBS and placed in a shaker at 37°C and 120 rpm. At pre-determined time points, samples were collected, adsorbed water was removed using cotton, and they were dried for up to a constant weight (*A*_t_). The degradation ratio was assayed using Equation (1):

(1) $$\mathrm{Degradation}\;\mathrm{ratio}=\frac{\left(A_{0}-A_{t}\right)}{A_{0}}$$

#### Mechanical properties of hydrogels

The compressive mechanical properties of hydrogels (diameter = 10 mm; height, 8 mm; *n *=* *4 for each group) were measured with a universal material testing machine (Instron 5567, Norwood, MA). Samples were analyzed for up to the rupture. The compressive strength was determined at a fixed strain rate of 10 mm/min by the maximum compressive strength of the strain–stress curves. Young’s modulus (*E*) was calculated from the stress–strain curves as the slope of the initial 10% linear region [28].

### Biocompatibility and biological function of hydrogels *in vitro*

#### Cytocompatibility

HUVECs were cultured in DMEM, while rBMSCs and MC3T3-E1 cells were cultured in alpha minimum essential medium (α-MEM, supplemented with 10% FBS, 100 U/ml penicillin, 0.1 mg/ml streptomycin) in an incubator at 37°C and 5% CO_2_. About 200 μl solutions of GM or GM@PRP (*n *=* *3) were added into 48-well cell culture plates and crosslinked by using 405 nm light for 1 min to achieve cured gels. Thereafter, HUVECs, rBMSCs, or MC3T3-E1 (5 × 10^4^ cells/well) were seeded along with the hydrogels. At pre-determined time points, the culture medium was removed and cell proliferation was evaluated using cell counting kit-8 assay (CCK-8, C0038, Beyotime, China) following manufacturer’s instructions. At Days 3 and 5, live/dead staining was performed and hydrogels were observed by using a fluorescence microscopy (DMi 8, Leica, Germany).

#### Scratch wound healing assay in vitro

The effect of PRP on cell migration was performed by scratch wound healing assay *in vitro*. The HUVECs (1 × 10^5^ cells/well) were evenly seeded into a 24-well cell culture plate and cultured for up to 24 h to afford a confluent cell monolayer [29]. Afterwards, scratches were created by 200 µl of autoclaved pipette tip. The conditioned medium was prepared from different types of hydrogels (diameter, 10 mm; length, 5 mm) by soaking them in 5 ml of 1% FBS-supplemented low-serum medium for up to 24 h. Approximately, 500 μl of the conditioned medium was added into respective wells (*n *=* *3). PRP group (PRP = 100 μl and DMEM = 900 µl) DMEM (1 ml) was used as control groups. The images of cell migration were collected by the optical microscope (TS100, Nikon, Japan) at 0 and 24 h. Quantitative analysis was performed with Image J software (NIH, v1.8.0, USA) to determine the area of remaining wound scratch. The cell migration rate was calculated by Equation (2):

(2) $$\mathrm{Migration}\;\mathrm{rate}\;\left(\%\right)=\frac{{(B}_{0}-B_{t})}{B_{0}}\times 100\%,\;$$

where *B*_0_ and *B*_t_ represent the area of scratch at *t *=* *0 and at pre-determined time point after cell migration.

#### Tube formation *in vitro*

Matrigel™ (catalog no. 356234, BD Matrigel, USA) was prepared and preserved overnight at 4°C. A total 100 μl of Matrigel was added into a pre-cooled 96-well plate and incubated at 37°C for up to 30 min [30]. One hundred microliters of HUVECs (1.0 × 10^4^ cells/well) were seeded into Matrigel™ along with 50 µl of the extract solution obtained from various hydrogels (*n *=* *3). After 6 h of incubation, the formation of capillary-like networks was observed by using an optical microscope. For quantitative analysis of angiogenic parameters, such as number of circles and nodes, images were analyzed by Image J software (NIH, v1.8.0, USA).

#### Transwell migration assay *in vitro*

The sterile GM or GM@PRP hydrogels (diameter = 10 mm; thickness = 2 mm) were added into wells in a cell culture plate (*n *=* *3). About 500 μl of serum-free medium was added into each well and samples were incubated for up to 2 h. Thereafter, 300 μl of cell suspension (2 × 10^4^ cells per insert) was added into the inserts and the cell culture plate was incubated at 37°C and 5% CO_2_ [31]. At 24 h, inserts were washed with PBS three times, and cells were fixed by using 500 μl of 4% paraformaldehyde (PFA) for up to 20 min. Next, 500 µl of crystal violet solution was added into the inserts and microwells for 15 min. The upper part of the membrane was carefully wiped with a cotton swab, while the cells migrated towards the lower side of the membranes were observed using an optical microscope. For quantification of cell migration, inserts were treated with 5% oxalic acid solution and optical density (OD) was measured at 590 nm.

#### Differentiation of rat BMSCs

To clarify the differentiation potential of hydrogels, the flow cytometry assay (BD LSRFortessa, BD Biosciences, CA, USA) was performed for the rBMSCs isolated from SD rats. The fluorescent markers were CD29 (fibronectin receptor), CD90 (glycophosphatidylinositol-linked glycoprotein), CD44 (stem cell marker), CD45 (hematopoietic stem cell marker), CD34 (vascular glycosylated transmembrane protein), CD11b-c (macrophages marker), IgG2a (cellular immune response receptor), IgG1 (cellular immune response receptor), and Armenian Hamster IgG (cellular immune response receptor). All of the antibodies for flow cytometry were from the MSC (rat) surface labeling assay kit (OriCell^®^, RAXMX-09011, T211009D501, Guangzhou, China).

#### ALP and ARS activity

The osteogenic differentiation of rBMSCs treated with the osteogenic induction medium (containing 10 mmol/l sodium β-glycerophosphate, 0.05 mmol/l vitamin C, 100 mmol/l dexamethasone, and α-MEM medium containing 10% FBS) of various groups was assessed by ALP and ARS assays. The rBMSCs (1 × 10^4^ cells/well) were inoculated into a 48-well cell culture plate containing 500 μl of α-MEM culture medium (supplemented with 100 U/ml penicillin, 0.1 mg/ml streptomycin, and 10% FBS). At 24 h, a total 500 μl of extract solution was added into each well and cells were cultured for up to Days 7 and 14. For ALP testing, cells were lysed with Western and IP cell lysate, while the collected supernatant was evaluated using an ALP assay kit (Beyotime Biotechnology; P00131, Jiangsu, China) and BCA protein analysis kit (Beyotime Biotechnology; P0012S, Jiangsu, China) following manufacturer’s instructions. For ARS testing, samples were rinsed with DI water three times, and then immersed in 2% ARS solution for 10 min. Meanwhile, the ALP staining was performed with BCIP/NBT analysis kit (Beyotime Biotechnology; C3206, Jiangsu, China) following manufacturer’s instructions and samples were observed both using a digital camera (Canon, EOS M200, Tokyo, Japan) and an optical microscope. For quantitative analysis of ARS, 5% acetic acid was used to treat the culture plate and the OD values were recorded at 570 nm using microplate reader (Epoch, BioTek, Winooski, VT, USA).

#### Western-blot analysis

The expressions of nicotinamide adenine dinucleotide phosphate, collagen type 1 (Col-I), bone morphogenetic protein-2 (BMP-2), and osteopontin (OPN) were determined by western-blotting analysis. The rBMSCs were cultured along with the extract solution obtained from the hydrogels for up to Day 7. The total protein was extracted and the protein concentration was determined by using a BCA protein assay kit as described in the section ‘ALP and ARS activity’. The samples were separated using sodium dodecyl sulfate–polyacrylamide gel electrophoresis (SDS–PAGE) and transferred to polyvinylidene fluoride (PVDF) membranes. Moreover, signal intensities of the protein bands were quantified by using ImageJ software (National Institutes of Health, v1.8.0, USA).

#### Transcriptome analysis

The rBMSCs and HUVECs were respectively cultured with the α-MEM osteogenic induction medium and DMEM high sugar medium supplemented with 1% PRP (for PRP groups) or without PRP (control group) for up to Day 4. HUVECs treated without or along with PRP were represented as CH and PH groups, while BMSCs treated without or along with the PRP were represented as CB and PB groups, respectively (Supplementary Table S2). Thereafter, rBMSCs and HUVECs were collected, and the total RNA was extracted by using RNAprep Pure Plant Plus Kit (DP441, TIANGEN, Hangzhou, China) and purified by RNAClean XP Kit (A63987, Beckman Coulter, Hangzhou, China) and RNase-Free DNase Set (79254, Qiagen, Hangzhou, China). Libraries were constructed using U-mRNAseq Library Prep Kit (AT4221, KAITAI-BIO, Hangzhou, China) with Ribo-off rRNA Depletion Kit (Bacteria, N407, Vazyme, Hangzhou, China), and were pooled and sequenced using the Illumina NovaSeq machine as 150-bp paired-end sequencing reads. For results assay, the GO enrichment analysis (Gene Ontology, http://geneontology.org/) showed the biological functions of differentially expressed messenger RNA (mRNAs), and was performed for all differentially expressed mRNAs. KEGG (Kyoto Encyclopedia of Genes and Genomes, http://www.kegg.jp/) is a database resource that contains a collection of manually drawn pathway maps representing our knowledge of the molecular interaction and reaction networks. By employing a method similar to the GO enrichment analysis, significantly enriched KEGG pathways were identified. The MiSeq sequencing and library construction were performed by technical staff at Hangzhou KaiTai Bio-lab.

### Animal experiments

The animal study was approved by the Experimental Animal Ethics Committee of Shandong Provincial Hospital and the ethical approval number for this animal study is No. 2022-115.

#### Subcutaneous implantation of hydrogels

Hydrogels (diameter = 8 mm and height = 3 mm) were prepared and subcutaneously (s.c.) implanted into SD rats (*n *=* *3 for each group) [32]. By week 4, blood samples were collected from eyes immediately after the intraperitoneal (i.p.) injection of 10% chloral hydrate; hepatorenal function was tested using an automatic biochemical analyzer (AU5800, Beckman, CA, USA). The implanted hydrogels along with the surrounding tissues were explanted and fixedusing 4% PFA. The sections were observed using hematoxylin–eosin (H&E) and Masson’s trichrome (MT) staining. For visceral toxicity, different organs, such as heart, liver, spleen, lung, and kidneys of mice were harvested and analyzed using H&E staining. To assay the mass loss GM and GM@PRP hydrogels *in vivo*, the hydrogel together with the surrounding thin tissue layer was taken off and further weighed at the corresponding time point.

#### Repair of tibial bone defect in rats

The 24 male SD rats (weight = 190–210 g; age = 6 weeks) were randomly assigned into four groups to establish bone defects of various sizes (*n* = 3 for every group at the 4 or 8 weeks). As shown in Supplementary Table S3, animals with only bone defect without any treatment were used as controls. On the other hand, animals with defects of various sizes (defect diameter = 1, 2, and 4 mm) received GM@PRP hydrogels (Supplementary Figures S3A and S4). Under sterile conditions, animals were anaesthetized by an intraperitoneal (i.p.) injection of 10 wt% chloral hydrate (dose = 300 mg kg^−1^). The skin of the right posterior supporting leg was removed and a longitudinal incision (length = 1.5 cm) was performed at the lateral end of the tibia with an iodophor disinfecting cloth. The tibia was dissected with an electric saw to form bone defects of various sizes (diameter = 1, 2, or 4 mm) (Supplementary Figure S3B). After ensuring good alignment, plate fixation was performed to remove most of the periosteum tissue in the defect area. Since GM@PRP hydrogels displayed adhesive properties, the corresponding solution was injected into the defect area followed by the photo-crosslinking with 405 nm visible light to completely fill the defect site for GM@PRP-1, GM@PRP-2, and GM@PRP-4 groups. Finally, the flap covers were closed and the skin was sutured to close the cortical window.

At 4- and 8-week post-operative procedures, the tibia was harvested and fixed with 4% PFA. Micro-computed tomography (μCT) was performed to analyze the degree of bone repair of the defects treated with various groups. The explanted bone tissues were decalcified, embedded in paraffin and sectioned. Histological analysis was performed with H&E and MT staining. Moreover, sections were analyzed by immunofluorescence (IF) staining for Col-I (type 1 collagen), osteocalcin (OCN), OPN, endothelial cell adhesion molecule-1 (CD31), and alpha-smooth muscle actin (α-SMA). The quantitative analysis was carried out using Image-Pro Plus software (*n* = 3 for each group). To discern bone regeneration, the tibia and fibula were simultaneously removed and the bone tissue was peeled off to expose the residual soft tissue; only the bone components were retained and the mass was quantified at the pre-determined time points.

### Statistical analysis

The experimental data were represented as mean ± SD. Independent samples *t*-test was used to compare differences between two group, while one-way analysis of variance was used to determine the level of significance for more than two groups. *P < 0.05, **P < 0.01, ***P < 0.001.

## Results

### Preparation and physicochemical characterization of hydrogels

**Figure 2.**
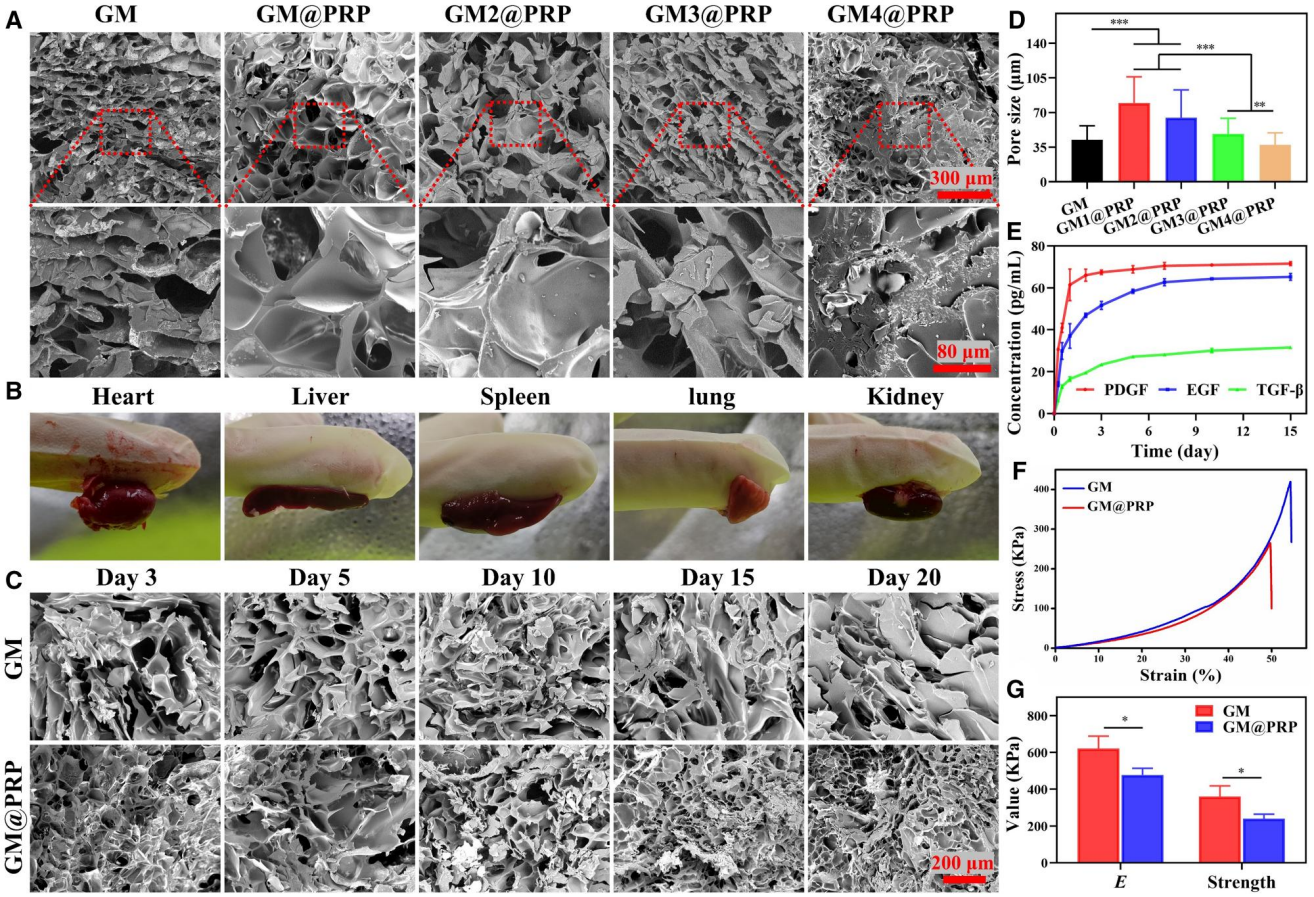
Preparation and physico-chemical characterization of hydrogels. **(A)** Morphological analysis of freeze-dried GM and GM@PRP hydrogels. Scale bars, 300 and 80 μm. **(B)** Representative images of GM@PRP hydrogels adhered to different types of organs, including heart, liver, spleen, lung, and kidney. **(C)** Morphological analysis of freeze-dried GM and GM@PRP hydrogels after *in vitro* degradation for up to different time points. Scale bar, 200 μm and applies to all of the images. **(D)** Pore sizes of hydrogels. **(E)** Sustained release of EGF, PDGF, and TGF-β1 from GM@PRP hydrogels immersed in the PBS *in vitro*. **(F)** Representative compressive stress–strain curves. **(G)** Young’s modulus and maximum compressive strength of hydrogels. *P < 0.05, **P < 0.01, and ***P < 0.001.

We prepared GM@PRP hydrogels by blending appropriate proportions of PRP and GM solutions. The PRP was collected from the blood while GM was obtained by the modification of Gel [10]. The GM solution displayed a transparent color. On the other hand, PRP was appeared to be of light-red color owing to the presence of fibrinogen, platelets and other components. The mixture of GM and PRP exhibited turbidity, which was also apparent in photo-crosslinked hydrogels (Figure 2). Moreover, GM is a temperature-sensitive hydrogel often prepared at 5–10 wt%. However, the GM may be prone to form uncross-linked hydrogels at temperatures below 22°C. We have used 50°C to better dissolve the GM hydrogel. The higher temperature may also maintain the physical state of the prepared GM hydrogel as a solution, which may be convenient for the subsequent preparation of GM@PRP hydrogel solution by simple mixing with the PRP. To better mix GM and PRP, the mixed solution of GM and PRP hydrogel was vortexed for up to 5 min.

The pore size of hydrogel was in the range of 10–200 μm. While pore size more than 40 um may be conducive for cell infiltration, pore size greater than 120 μm may adversely affect the mechanical properties of hydrogels [33, 34]. The GM hydrogels displayed a sheet-like structure, while GM/PRP hydrogels exhibited a porous structure (Figure 2A). Nevertheless, both the average pore diameter and number of pores were decreased with an increase in the GM content in the hydrogels. Pore size of the GM was significantly increased from 42.4 ± 14.2 μm to 79.7 ± 26.3 μm in GM@PRP hydrogels, while it was decreased to 64.8 ± 28.0 μm, 48.4 ± 15.7 μm, and 37.2 ± 12.4 μm for GM2@PRP, GM3@PRP, and GM4@PRP hydrogels, respectively (Figure 2D). In the GM and GM@PRP groups, the concentration of GM was consistent, and the pore size distribution was significantly different due to the introduction of PRP. The incorporation of PRP led to a certain decrease in the crosslinking of the hydrogels. Moreover, the GM content was increased with a decrease in the PRP content. Consequently, the cross-linking degree was higher in the hydrogels with the lower content of PRP alongside a decreasing trend of the pore size. These data showed good morphology and pore size of GM@PRP hydrogels, which have similar ratio of GM and PRP. Therefore, GM@PRP hydrogels (i.e. GM = 10 wt%) were used for the subsequent experiments.

The adhesion of GM@PRP hydrogels was assessed using different types of organs, including heart, liver, spleen, lung, and kidneys. These organs as well as bone were tightly adhered to the hydrogels and did not fall off vertically for up to 30 s. The injectable hydrogels filled into bone defects were also found to be stable, which did not fall off, which is indicative of the significant adhesion properties of the hydrogels (Figure 2B and Supplementary Figure S6). Lap-shear testing further revealed good adhesion strength of GM and GM@PRP hydrogels; the values for the lap-shear strength were found to be 34.8 ± 4.6 and 22.9 ± 4.1 kPa for GM and GM@PRP hydrogels, respectively. The GM showed significantly higher adhesion strength than that of the GM@PRP hydrogel which may be ascribed to the reduced crosslinking mediated by the PRP (Supplementary Figure S6A and B). Moreover, the tissue surfaces such as bone and skin possess amino groups (–NH_2_), carboxylic groups (–COOH), hydroxyl groups (–OH), and sulfhydryl groups (–SH). The GelMA also exhibits the above-mentioned functional groups alongside methacrylic (MA) groups. During photo-crosslinking of hydrogels in the presence of a photo initiator and a light source, the MA groups may enable crosslinking of the hydrogels. During crosslinking, the hydrogel surface may also react with the tissue surface through hydrogen bonding, thereby promoting tissue adhesion.

GM and GM@PRP hydrogels were further subjected to an *in vitro* degradation followed by morphological analysis with SEM (Figure 2C). After degradation, the remained mass was found to be 66.0 ± 4.6% and 57.6 ± 5.1% (Day 20) and 48.1 ± 4.5% and 39.1 ± 2.6% (Day 35) for GM and GM@PRP hydrogels, respectively (Supplementary Figure S7). The degradation of hydrogels may be ascribed to both the GM and PRP [3]. Morphological assessment did not reveal a significant change between GM and GM@PRP hydrogels after degradation *in vitro* plausibly owing to the low swelling rate and longer degradation cycles. The mechanical properties of the hydrogel were also well-maintained without obvious fragments, thereby indicating that the degradation may have gradually occurred from the outside to the inside with negligible morphological changes.

The PRP is rich in diverse GFs, whose release may have implications for the biological functions of the hydrogels. Consequently, we collected the supernatant after the degradation of scaffolds for up to different time points *in vitro* and carried out ELISA for PDGF, EGF, and TGF-β1 [10]. The above-mentioned GFs showed marked release at Day 5, which became almost constant thereafter. The cumulative release of PDGF, EGF, and TGF-β1 from GM@PRP hydrogels was 71.6 ± 1.0 pg/ml, 65.2 ± 1.6 pg/ml, and 31.6 ± 0.5 pg/ml at Day 15 *in vitro*. The content of various GFs may differ in the PRP, which may lead to their differential release. We observed significantly less release of TGF-β1 than that of the PDGF and EGF from GM@PRP hydrogels [35, 36]. While we performed release studies in in a dynamic environment *in vitro*, it may be different than that of the *in vivo* microenvironment. These data further demonstrate the effectiveness of GM@PRP hydrogels for the sustained release of different types of GFs [10]. Thus, these results inform about the sustained release of GFs from GM@PRP hydrogels *in vitro*.

Appropriate mechanical properties of scaffolds may be beneficial for maintaining the integrity of the scaffolds as well as supporting cell adhesion and proliferation [37]. Representative compressive strain–stress curves revealed that the hydrogels can be compressed for up to 50% of their volume (Figure 2F). The incorporation of PRP into hydrogels decreased their mechanical properties (Young’s modulus: GM@PRP, 478.2 ± 36.2 kPa and GM, 621.4 ± 67.7 kPa; maximum compressive stress: GM@PRP, 239.7 ± 24.6 kPa and GM, 360.5 ± 58.0 kPa). It is worthy to note that the GM@PRP hydrogel displayed reasonable mechanical properties to serve as scaffolds for bone regeneration [38].

### Biocompatibility and biological function *in vitro*

**Figure 3.**
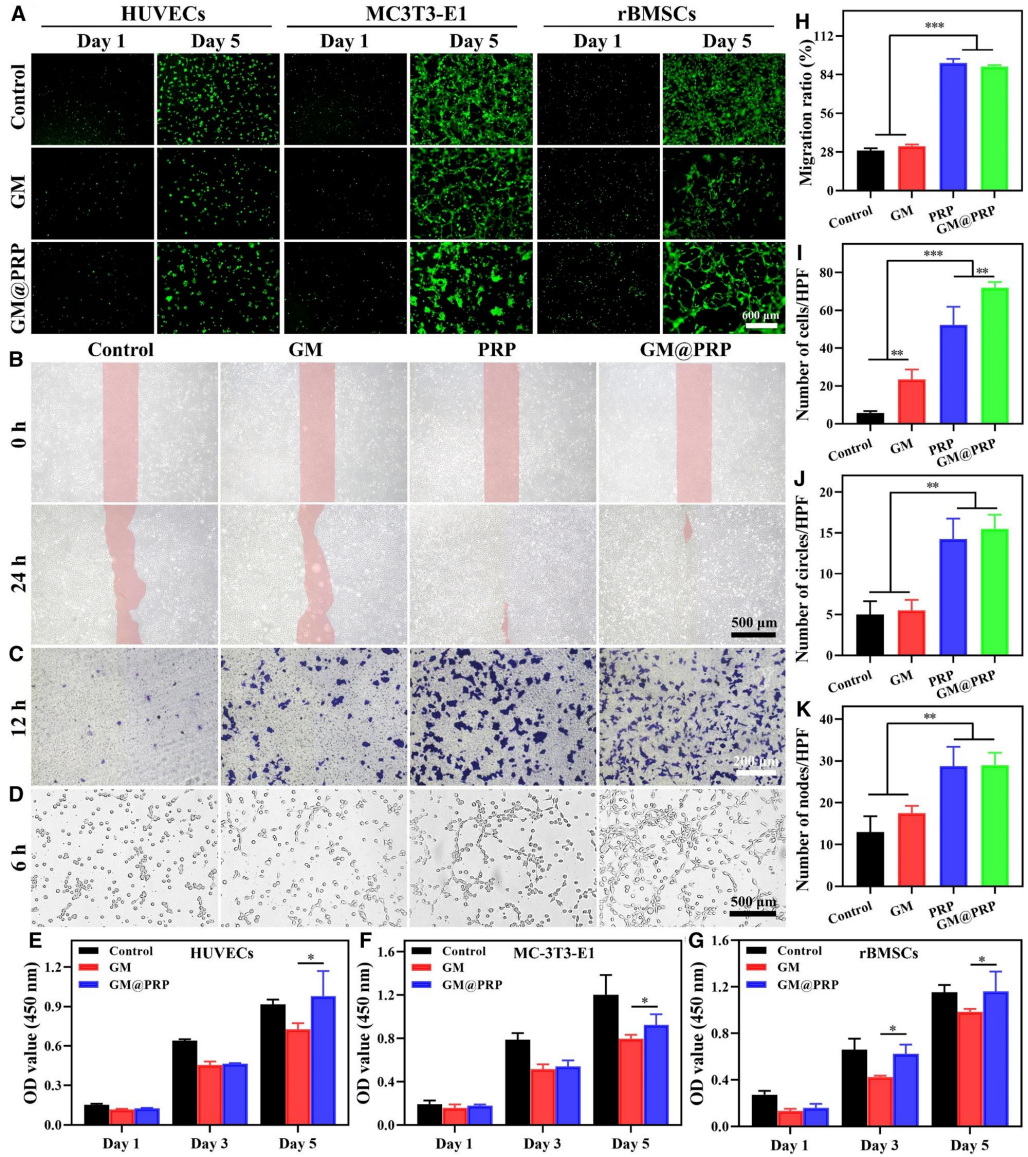
Biocompatibility and biofunction of hydrogels *in vitro*. **(A)** Live/dead staining of HUVECs, MC3T3-E1, and rBMSCs at Days 1 and 5. Scale bar, 600 μm and applies to all the images. **(B)** Representative images in a scratch wound healing assay for up to 24 h. Scale bar, 500 μm. **(C)** Representative images of the migration of HUVECs in a transwell migration assay *in vitro* at 12 h. **(D)** Tube formation of HUVECs for up to 6 h. The CCK-8 assay of HUVECs **(E)**, MC3T3-E1 **(F)**, and rBMSCs **(G)** at Days 1, 3, and 5. Cell migration ratio in a scratch wound healing assay **(H)**, migrated cells in a transwell migration assay **(I)**, number of nodes **(J)**, and number of circles **(K)**. *P < 0.05, **P < 0.01, and ***P < 0.001.

The cytocompatibility of scaffolds was next deciphered by assessing cell viability and proliferation (Figure 3). Live/dead staining manifested negligible difference between GM and GM@PRP hydrogels in terms of the viability and morphology of different types of cells, such as HUVECs, MC3T3-E1, and rBMSCs (Figure 3A). While all groups showed only a few numbers of cells at Day 1, sufficiently more number of cells were observed at Day 5. Moreover, cell proliferation assay by CCK-8 showed a continuous increase in cell proliferation over time in all the groups, thereby further suggesting the cytocompatibility of scaffolds (Figure 3E–G). Both the GM and GM@PRP hydrogels showed less OD values compared to the control group. Of GM and GM@PRP, the latter showed significantly higher OD values, which may be attributed to the beneficial effect of PRP for cell proliferation [39].

The chemotactic effect of PRP was discerned using a scratch wound healing assay *in vitro*. As can be observed from Figure 3B, initially there was almost similar wound depth, which had become narrower in the PRP and GM@PRP groups. The values for the cell migration were 28.9 ± 1.7%, 32.2 ± 1.2%, 92.2 ± 3.0%, and 89.7 ± 1.1% for control, GM, PRP, GM@PRP groups at 24 h (Figure 3H). Transwell migration assay was used to verify the chemotactic response of the PRP to the HUVECs; the PRP had a significant role for promoting cell migration. Quantitative analysis revealed that the number of migrated cells were 5.75 ± 0.96, 23.5 ± 5.2, 52.3 ± 9.6, and 72.0 ± 2.9 per HPF for control, GM, PRP, GM@PRP groups, respectively at 12 h (Figure 3C and I). Transwell migration assay was also carried out to evaluate the migration of HUVECs either by only using the cell culture medium (control group) or the extract solution of GM, PRP, and GM@PRP hydrogels. At 12 h, PRP and GM@PRP showed significant migration of HUVECs than that of the control and GM groups (Figure 3C and I). Intriguingly, the incorporation of PRP also had a beneficial angiogenic effect as revealed by more numbers of angiogenic parameters (i.e. number of nodes and circles) in PRP and GM@PRP groups as compared to the control and GM groups (Figure 3D, J, and K). These data indicate that the PRP has chemotactic abilities, which may not only promote the migration of HUVECs but may also stimulate the formation of angiogenic networks [40, 41].

**Figure 4.**
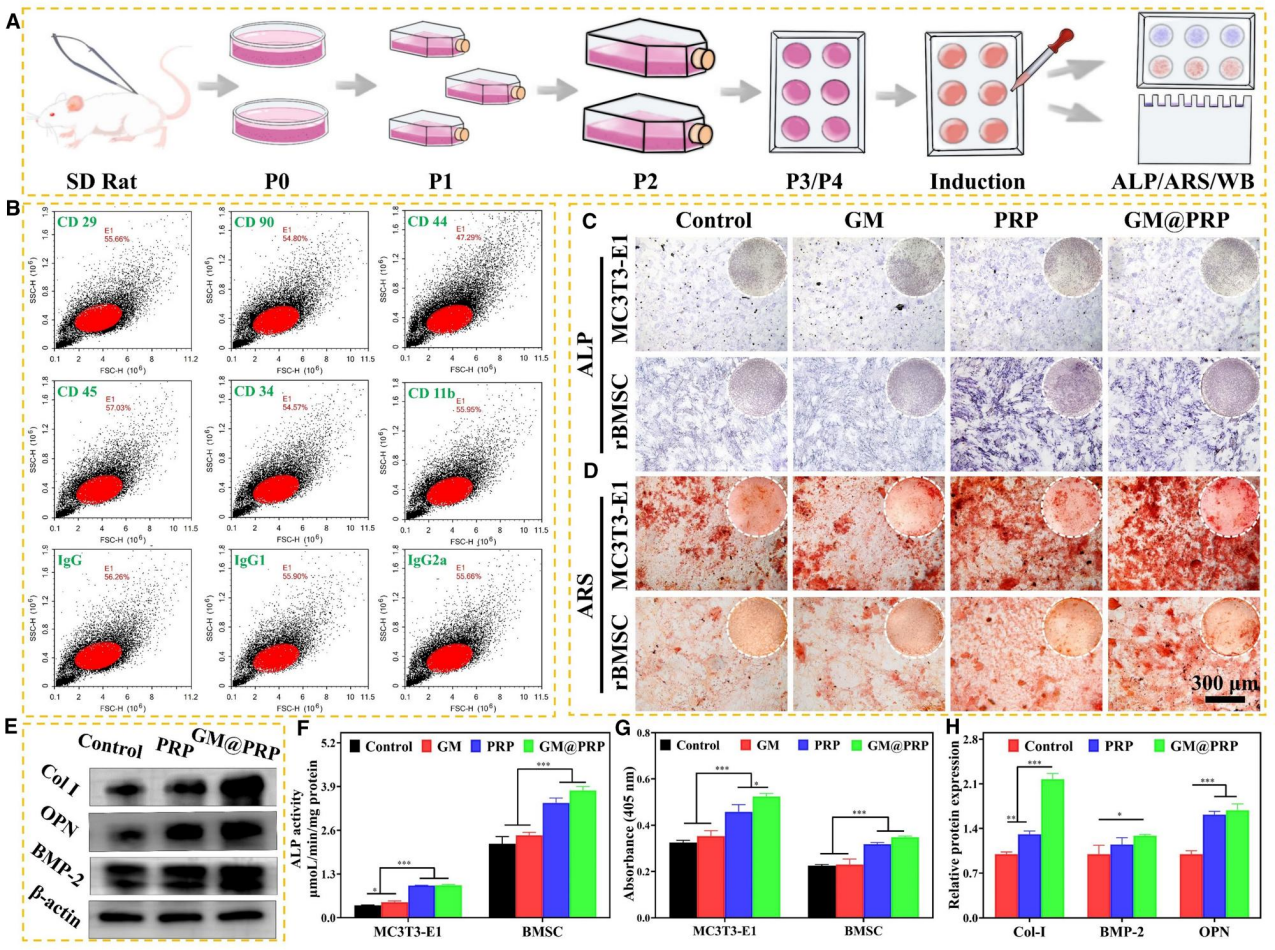
Osteogenic differentiation of MC3T3-E1 and rBMSCs *in vitro*. **(A)** Schematic representation of osteogenic assay. **(B)** Flow cytometry assays of rBMSCs for expression of various surface antigens, including CD29, CD90, CD44, CD45, CD34, CD11b, IgG, IgG1, and IgG2a. Microscopic images for ALP staining **(C)** and ARS staining **(D)** of MC3T3-E1and rBMSCs cultured along with the extract solution of different types of hydrogels at Day 14. Scale bar, 300 μm. Western-blot analysis for Col-I, OPN, and BMP-2 in rBMSCs cultured with the extract solution of the hydrogels at Day 5 **(E)**. Quantitative analysis of ALP activity **(F)** and ARS staining **(G)** of MC3T3-E1 and rBMSCs at Day 14. (**H**) Quantitative analysis of western blot for different types of osteogenic proteins (including Col-I, BMP-2, OPN) in rBMSCs after osteogenic induction at day 5. *P < 0.05, **P < 0.01, and ***P < 0.001.

The osteogenic differentiation of rBMSCs was also performed *in vitro* by evaluating the expression of various cell markers, including CD29, CD90, CD44, CD45, CD34, CD11b-c, IgG2a, IgG1, and IgG by using flow cytometry (Figure 4). As shown in Figure 4B and Supplementary Figure S8, the cell surface-specific antigens, such as CD29, CD90 and CD45, which are associated with stem cell phenotype showed positive expression (> 90%), indicating the high purity of stem cells and possibly their good differentiation potential. The rBMSCs exhibit multi-directional differentiation potential. Vascular-related surface antigens (CD45 and CD34) were negative (<1%), while the expression of macrophage-associated antigen (CD11b-c) was less than 1%, thereby indicating fewer chances for the differentiation of rBMSCs towards macrophages. Similarly, the expressions of immune-related antigens, such as IgG2a, IgG1, and IgG were less than 0.5% [42].

Both the MC3T3-E1 and rBMSCs were induced with an osteogenic medium and osteogenic differentiation was studied by performing ALP staining and ARS staining at Day 14. For both cell types, PRP and GM@PRP groups displayed significantly higher ALP activity as well as the formation of calcium nodules than that of the control and GM groups (Figure 4C and D). Quantitative analysis also showed significantly higher ALP activity and absorbance for ALS staining as compared to control and GM groups (Figure 4F and G). Western-blot analysis was performed to evaluate the expression of different types of osteogenic proteins, such as Col-I, OPN, and BMP-2 for the rBMSCs-loaded hydrogels (Figure 4E). The PRP and GM@PRP groups showed significantly higher expression of Col-I, OPN and BMP-2 than that of the control group (Figure 4H). These results inform about the potential of the PRP to promote the osteogenic differentiation and mineralization in MC3T3-E1 and rBMSCs.

### Transcriptome analysis of HUVECs and rBMSCs

**Figure 5.**
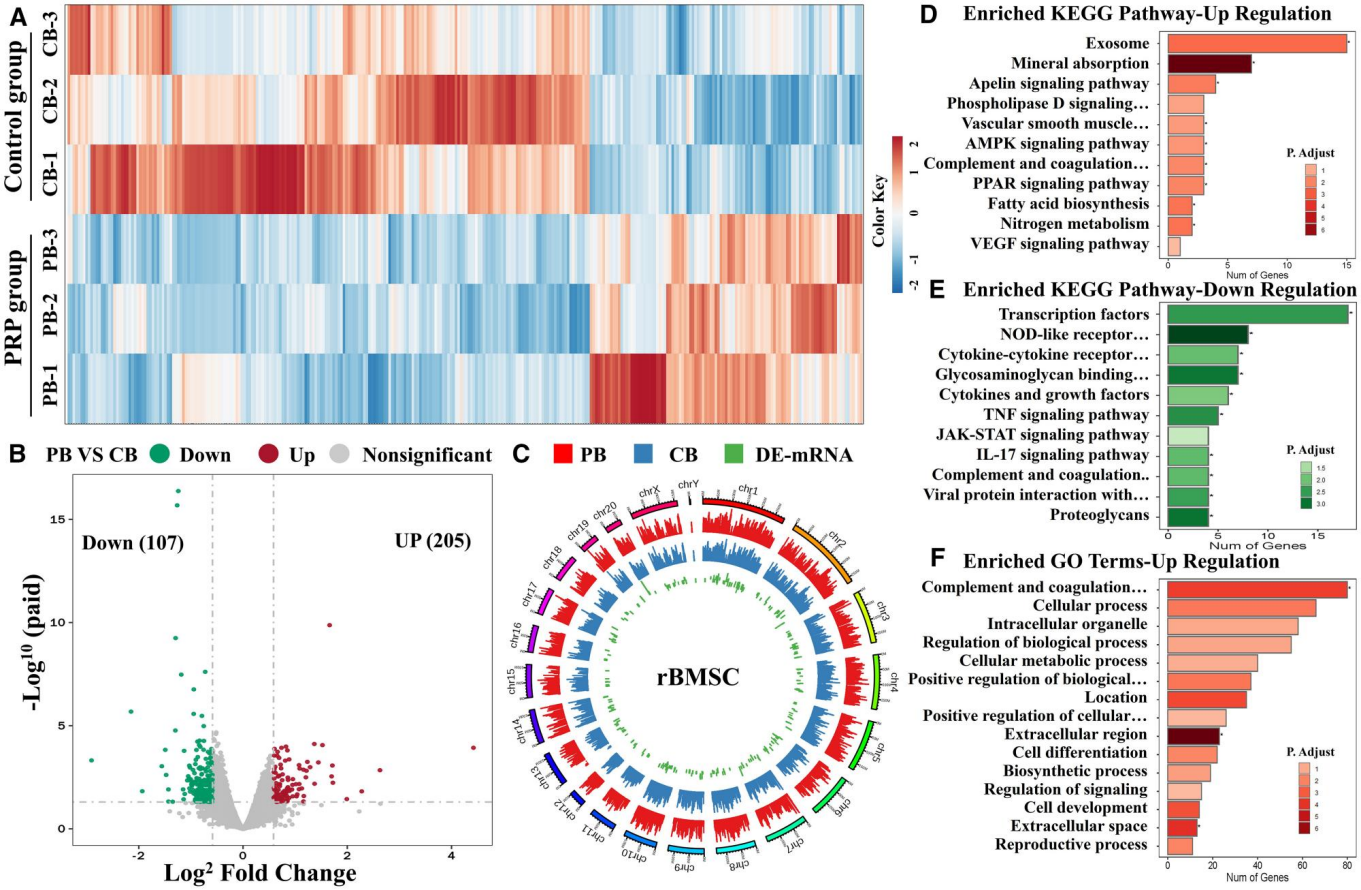
Whole transcriptome RNA sequencing of rBMSCs col-cultured along with PRP at Day 4. PB (rBMSCs treated with PRP) and CB (rBMSCs treated without PRP). **(A)** Heat map analysis of the top 100 differentially expressed genes (DEGs) ranked by log2FC values in CB and PB groups. **(B)** Volcano plot of DEGs and **(C)** differential gene genome circle map of CB and PB groups. Kyoto encyclopedia for genes and genomes (KEGG) pathway enrichment analysis of the upregulated genes **(D)** and downregulated genes **(E)**. gene ontology (GO) enrichment analysis of the upregulated genes **(F)**. *P < 0.05, **P < 0.01, and ***P < 0.001.

**Figure 6.**
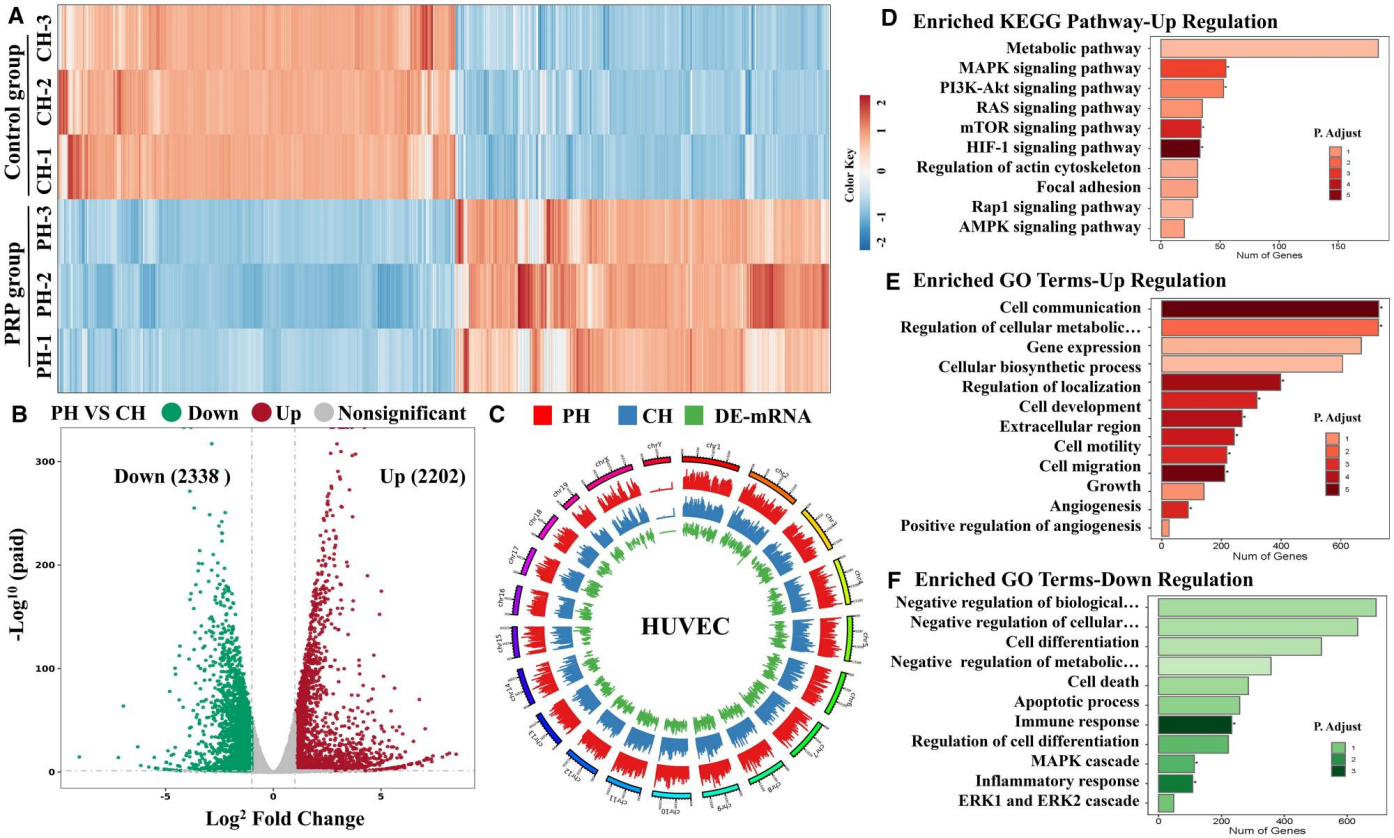
Whole transcriptome RNA sequencing of HUVEC treated with PRP co-culture compared with the control group to reveal transcriptome difference at Day 4. PH (HUVEC treated with PRP) versus CH (HUVEC treated without PRP). **(A)** Heatmap analysis of the top 100 DEGs ranked by log2FC values between control and PRP groups. **(B)** Volcano plot of DEGs and **(C)** differential gene genome circle map between control and PRP groups. **(D)** KEGG pathway enrichment analysis of the upregulated genes. GO enrichment analysis of the upregulated genes **(E)** and downregulated genes **(F)**. *P < 0.05, **P < 0.01, and ***P < 0.001.

We next performed transcriptomics analysis to explore regulatory effects of PRP for vasculogenic and osteogenic differentiation of HUVECs and rBMSCs, respectively (Figures 5 and 6). The complex heatmap showed the DEGs with the top 100 log2FC values on differentially expressions of these genes on PB and CB groups after difference analysis (Figure 5A). The volcano plot in Figure 5B showed an upregulation of 205 genes, while a downregulation of 107 genes. The gene genome circle map also displayed an existence of the large number of DEGs expressions. These data showed that the PRP co-culture treatment could alter gene expression in rBMSCs (Figure 5B and C).

The KEGG pathway enrichment analysis revealed remarkable potential of the PRP group to activate and upregulate metabolism-related signaling pathways (e.g. exosomes, nitrogen metabolism, fatty acid biosynthesis, peroxisome proliferator-activated receptor signaling pathways, phospholipase D signaling pathway, etc.), vascular-related signaling pathway (e.g. apelin signaling pathway, vascular smooth muscle contraction, VEGF signaling pathway, etc.), and mineral-related signaling pathway (mineral absorption). On the other hand, KEGG pathway enrichment analysis indicated salutatory effects of the PRP group to suppress interleukin-17, anus Kinase-Signal Transducers and Activators of Transcription (JAK-STAT), tumor necrosis factor alpha, and Nucleotide-Binding Oligomerization Domain-like receptor signaling pathway (Figure 5D and E). These pathways are related with the polarization of macrophages, activation of fibroblasts as well as aging and apoptosis of cells [30]. The GO analysis revealed an upregulation of biological, cellular, and biosynthetic processes as well as cell proliferation. Taken together, these results indicated that the PRP could promote the proliferative and metabolic capacity of cells, thereby potentially accelerating tissue regeneration (Figure 5F).

Similarly, the regulatory effects of PRP for HUVECs behaviors were investigated. The complex heatmap analysis showed the DEGs on PH and CH groups after difference analysis, indicating that the expression patterns of the PH-1, PH-2, and PH-3 groups were similar but notably distinct from the CH groups (Figure 6A). The volcano plot showed an upregulation of 2338 genes, while downregulation of 2202 genes. Gene genome circle map also displayed sufficient expression of DEGs (Figure 6B and C). As can be seen from enriched KEGG pathways, the PRP could up-regulate different types of signaling pathways, such as metabolically related signaling pathway (metabolic pathways, AMPK signaling pathway), cell proliferation and migration-related signaling pathway (e.g. MAPK signaling pathway, Ras signaling pathway, mTOR signaling pathway, Focal adhesion, Rap1 signaling pathway, etc.), and angiogenesis-related signaling pathway (PI3K-Akt signaling pathway, HIF-1 signaling pathway) (Figure 6D). Meanwhile, GO analysis showed that most of the genes related to cell proliferation, cell migration, cell metabolism, and angiogenesis were upregulated. Taken together, these results indicated that the changes in the HUVECs’ behavior may be due to the regulation of key genes related to the cell viability and angiogenesis (Figure 6E and F).

### Biocompatibility *in vivo*

**Figure 7.**
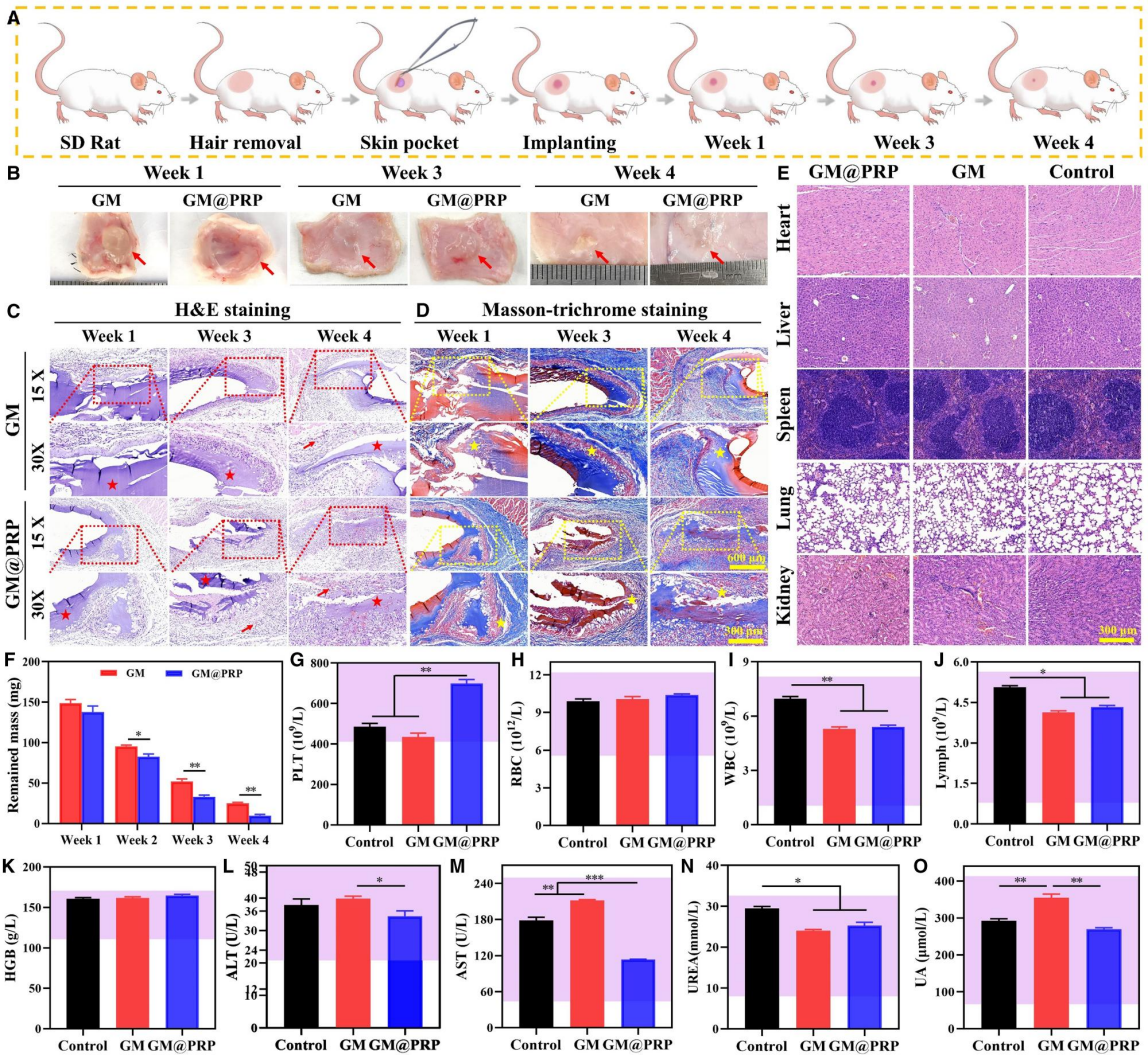
Biocompatibility of hydrogel *in vivo*. **(A)** Schematic representation of subcutaneous implantation of scaffolds for up to 4 weeks. **(B)** Digital photographs of GM and GM@PRP hydrogels at week 1, 3, and 4 after subcutaneous implantation. H&E staining **(C)** and MT staining **(D)** of explants and surrounding tissues. Scale bars, 600 and 300 μm. The arrows point toward infiltrating cells, while stars point towards hydrogel scaffolds. H&E staining of heart, liver, spleen, lung, and kidney. Scale bar, 300 μm. **(E)**. The remained mass **(F)** of hydrogels after subcutaneous implantation at different time points. Routine blood analysis for platelets (PLT) **(G)**, red blood cells (RBCs) **(H)**, white blood cells (WBCs) **(I)** and lymphocytes **(J)** of healthy rats treated with GM and GM@PRP hydrogels for 4 weeks. Blood biochemistry data, including hemoglobin (HGB) **(K)**, alanine aminotransferase (ALT) **(L)**, and aspartate aminotransferase (AST) **(M)**, urea (UREA) **(N)**, and uric acid (UA) **(O)** of healthy rats treated with hydrogels for 4 weeks. *P < 0.05, **P < 0.01, and ***P < 0.001.

To gain a further insight into the degradation of scaffolds, they were subcutaneously implanted into rats (Figure 7A). The photographs of GM and GM@PRP hydrogels revealed gradual degradation of the hydrogels with an increase in the implantation time (Figure 7B). The quantitative analysis further corroborated these results; GM@PRP exhibited significantly less remained mass than that of the GM group (mass remained 4 weeks post-implantation: GM, 25.0 ± 1.0 mg and GM@PRP, 9.7 ± 1.53 mg). H&E staining showed infiltration of various cell types into GM@PRP hydrogels, including neutrophils, fibroblasts, macrophages, and lymphocytes (Figure 7C). Masson’s trichrome staining showed that GM and GM@PRP hydrogels were gradually replaced with the neo-tissues, such as fibroblasts and granulation tissue with an abundant neovascularization alongside promoting collagen deposition. Moreover, the residual scaffold materials were observed in all groups (Figure 7D).

Major organs, such as heart, liver, spleen, lung, and kidney were also explanted and stained with the H&E. The explants did not reveal toxicity, thereby indicting the safety of the hydrogels (Figure 7E). Similarly, there was an insignificant difference among control, GM, and GM@PRP groups in terms of different types of blood cells, including platelets, red blood cells (RBCs), white blood cells (WBCs), and lymphocytes (Lymph) as well as blood biochemical analysis (i.e. HGB, ALT, AST, UREA, UA, etc.). To put together, these results indicated the safety and biocompatibility of the hydrogels (Figure 7G–O).

### Bone regeneration *in vivo*

**Figure 8.**
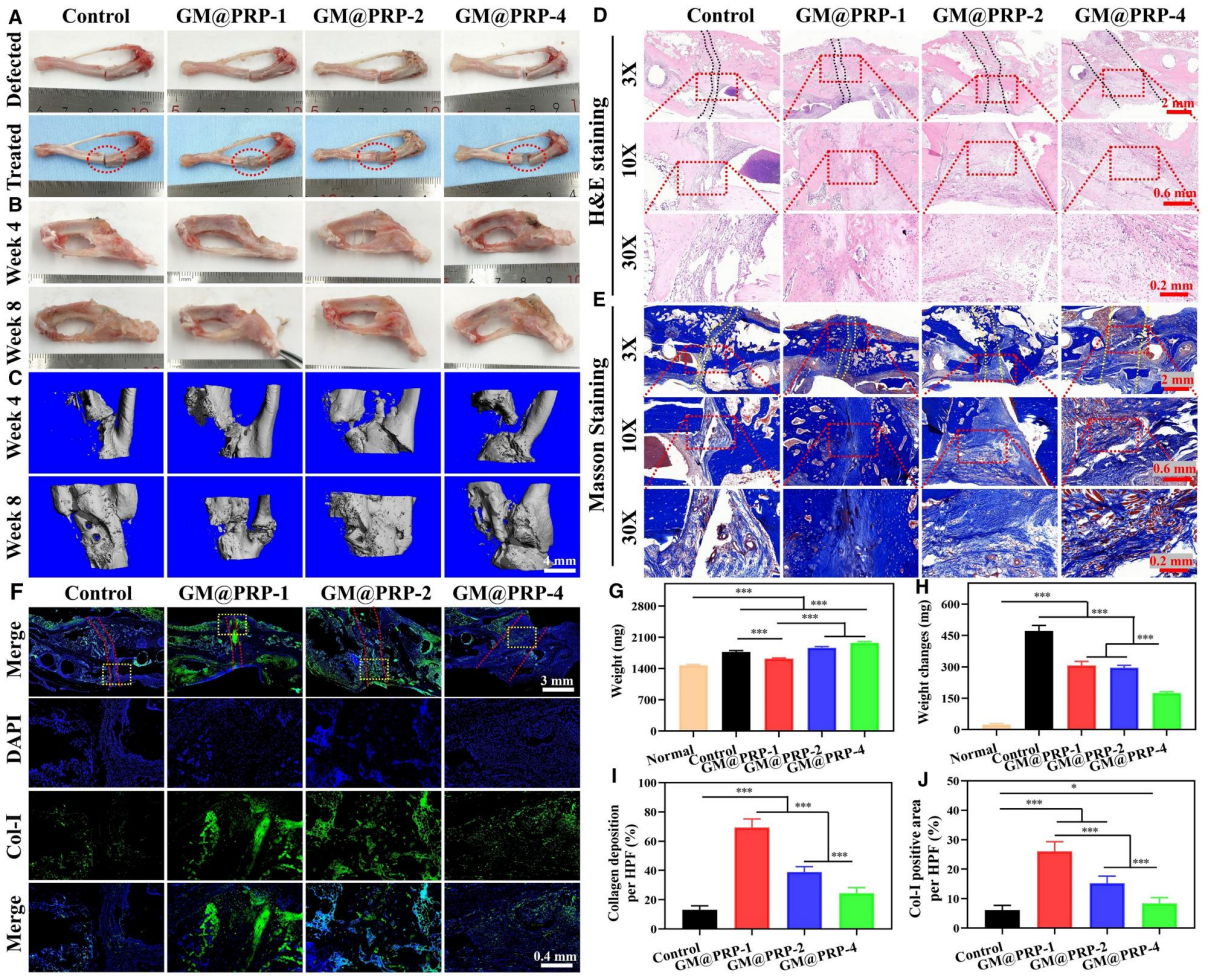
Evaluation of hydrogel scaffolds in a tibial defect model. **(A)** The digital pictures of treated and untreated tibial defects. **(B)** The digital pictures of bone defects treated with GM@PRP. **(C)** The CT photographs of regenerated bone defects at 4- and 8-week post-operatively. Scale bar, 4 mm. **(D)** H&E staining, **(E)** MT staining, and **(F)** Col-I staining of repaired bone defects 8-week post-operatively. Scale bars, 2 mm, 600 and 200 μm for MT staining, 3 mm and 200 μm for Col-I IF staining. Quantitative analysis of the tibial weight **(G)**, tibial weight change between 0–8 weeks **(H)**, collagen deposition **(I)**, and Col-I positive area per high power field (HPF) **(J)**. *P < 0.05, **P < 0.01, and ***P < 0.001.

Tibial defects are widely employed as large-sized bone injury models. Tibial defects of various sizes (e.g. 1, 2, and 4 mm, etc.) were established. Untreated (1 mm) defects were employed as controls, while 1, 2, and 4 mm defects treated with GM@PRP hydrogels were used as experimental groups and indicated as GM@PRP-1, GM@PRP-2, and GM@PRP-4, respectively (Supplementary Table S3). The control group had significant bone hyperplasia than that of the normal tibia and GM@PRP groups 8-week post-operatively (Supplementary Figure S9A and B). The deformation was increased with an increase in the size of bone defect; large-sized defects, including 2 and 4 mm were not conducive for bone regeneration. The micro-computed tomography (microCT) results displayed poor regeneration in the control and GM@PRP-1 groups (Supplementary Figure S9C and D). On the other hand, the 4 mm defects treated with GM@PRP still displayed an intense bone deformation despite sufficient tissue repair (Figure 8C). It is worth to note that the weight of the tibial bone tissues was significantly higher in the 2 and 4 mm defects treated with GM@PRP as compared to the other groups (weight of the tibial defect by Week 8 post-operatively: GM@PRP-2, 1865.3 ± 27.8 mg; GM@PRP-4, 1976.0 ± 28.2 mg; GM@PRP-1, 1620.3 ± 16.1 mg; control, 1773.5 ± 29.8 mg; and normal, 1475.5 ± 17.3 mg). These data indicated that the large-size bone defects may lead to bone hyperplasia and fibrosis. Meanwhile, the weight changes of the tibia were 24.0 ± 5.0 mg, 471.0 ± 26.9 mg, 306.3 ± 20.1 mg, 295.9 ± 11.7 mg, 173.9 ± 6.9 mg for healthy (normal group), control, GM@PRP-1, GM@PRP-2, and GM@PRP-4 groups, respectively. These data showed that the defects with the size less than 2 mm exhibited better osteo-inductivity than that of the other groups (Figure 8G and H).

H&E staining revealed unhealed bone defects in the control group. In contrast, the defects treated with GM@PRP hydrogels were infiltrated with the neo-tissue (Figure 8D and Supplementary Figure S10A). Bone defects showed a gradual increase in the number of fibroblasts. Moreover, the mineralization effect was significantly weakened away from the normal tibial tissues (Figure 8D and Supplementary Figure S10B). The MT staining showed relatively poor collagen regeneration in the control group than that of the GM@PRP-1, GM@PRP-2, and GM@PRP-4 groups, which all displayed significant collagen regeneration (Figure 8I; collagen deposition per HPF: GM@PRP-4, 24.3 ± 4.0%, GM@PRP-2, 38.9 ± 3.8%, GM@PRP-1, 69.3 ± 5.9%, and control, 13.0 ± 2.9%). Moreover, the MT staining showed new bone formation in the GM@PRP-1 groups, while other groups exhibited fibrous tissues at the implantation site. These results indicated that the GM@PRP hydrogels can effectively promote the regeneration of bone defects as well as induce collagen deposition (Figure 8E and J and Supplementary Figures S10 and S11). IF staining showed significantly higher Col-1 positive area in GM@PRP-1 and GM@PRP-2 groups as compared to the other groups (Figure 8F and J and Supplementary Figure S12).

**Figure 9.**
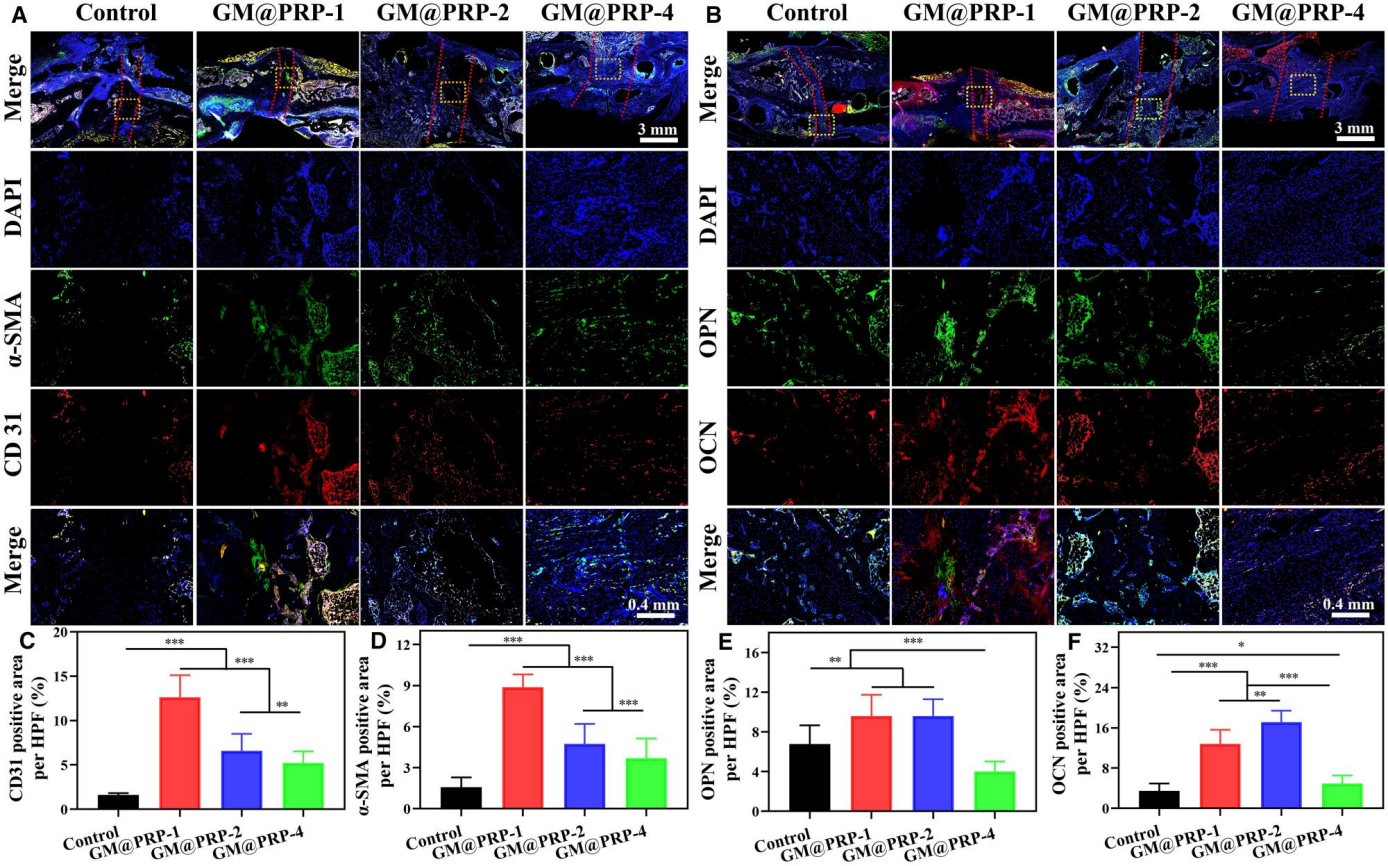
Osteogenic and angiogenic activity of hydrogels. IF staining of explants 8-week post-operatively. **(A)** IF staining for α-SMA and CD31 and **(B)** OCN and OPN. Scale bars, 3 mm and 400 μm. Quantitative analysis of CD31^+^**(C)**, α-SMA^+^**(D)**, OPN^+^**(E)**, and OCN^+^**(F)** areas. *P < 0.05, **P < 0.01, and ***P < 0.001.

Since blood vessels plays an important role for the diffusion of oxygen as well as the transport of nutrients, vascularization of the implants holds great promise for their *in vivo* performance [2]. The control group showed only a few numbers of CD31 and α-SMA positive cells (Figure 9A, C, and D; Supplementary Figure S13A). In striking contrast, the defects treated with the GM@PRP hydrogels showed remarkably higher CD31^+^ and α-SMA^+^ areas, thereby indicating a beneficial effect of the PRP on the vascularization of the scaffolds. Since large-sized defects may require longer time for vascularization, large defects (2 and 4 mm) showed relatively lower CD31^+^ and α-SMA^+^ positive areas (Figure 9A). The GM@PRP-1 and GM@PRP-2 groups also manifested an intense immunofluorescence staining for OCN and OPN than that of the control and GM@PRP-4 groups (Figure 9B). Quantitative analysis further corroborated these results; GM@PRP-1 and GM@PRP-2 groups showed significantly higher OCN^+^ and OPN^+^ areas as compared to the other groups. We surmise that the PRP may have a salutatory effect to promote biomineralization as well as the deposition of OCN and OPN (Figure 9E and F and Supplementary Figure S13B). Altogether, GM@PRP hydrogels showed a beneficial effect to promote the regeneration of segmental bone defects albeit their mild performance for the regeneration of the large bone defects. The large-sized bone defects may easily undergo bone hyperplasia, thereby lowering vascularization and osteogenic mineralization properties of hydrogels.

## Discussion

The treatment of large-sized bone defects is very important to maintain the physical integrity of the patient as well as guarantee normal physiological function [38, 43]. However, there is currently no uniform standard for the diagnosis and classification of bone defects alongside a lack of the consensus on the definition of large-sized bone defects. It has been suggested that the bone defects with a length of more than 1.5 times than that of the bone diameter, or a defect length (>1 cm) and a cortical bone diameter loss (>50%) are considered as large-sized bone defects. In this study, we used 6 weeks old SD rats. The length of the tibia was 3.64 ± 0.11 cm, while the diameters of the tibia from the D1 to D4 were 2.67 ± 0.48 cm (D1), 2.34 ± 0.41 cm (D2), 3.15 ± 0.55 cm (D3), and 4.28 ± 0.65 cm (D4) (Supplementary Figure S2 and Supplementary Table S4). We have chosen the region between D2 and D3 (diameter = 2.5 mm) to prepare tibial defects of various dimensions (defect size = 1, 2, and 4 mm). The 4-mm tibial defects were chosen as large-sized bone defects.

Different types of strategies have been employed for the repair of large-sized bone defects [44, 45]. For irregular-shaped bone defects, scaffolds should fill the site of the bone defect as well as simultaneously induce osteogenesis and angiogenesis [46–48]. Very recently, scaffolds loaded with the autologous cells have been shown to promote bone tissue repair [49, 50]. The PRP from autologous blood components has been shown to facilitate tissue repair in multiple injury models presumably due the synergistic effect of different types of GFs, such as PDGF, EGF, VEGF, and TGF-β1. These GFs may promote the migration, proliferation, and differentiation of different types of cells, including endothelial cells, fibroblasts, and smooth muscle cells [51]. Meanwhile, VEGF and TGF-β1 may also play a pivotal role for the production of the ECM and the formation of blood vessels [10, 52].

The PRP contains fibrinogen, which could form hydrogels in the presence of pro-thrombin and metallic ions (e.g. calcium ions (Ca^2+^)). Nevertheless, relatively poor mechanical properties of these hydrogels as well as rapid release of bioactive factors from them may hamper an effective utilization of PRP for TE applications [7]. Parallelly, platelets in the PRP, if activated, may have a very short half-life, thereby further reducing an effectiveness of PRP *in vitro* and *in vivo* [10]. The GM has been intensively harnessed for regenerative medicine and TE applications owing to its excellent biocompatibility, biodegradability, low immunogenicity, and cost-effectiveness. Moreover, GM has been shown to promote osteogenic differentiation of MSCs [53]. We leveraged photo-crosslinked GM@PRP hydrogels to furnish osteo-inductive scaffolds, which may also enable spatiotemporal release of bioactive factors [11, 54]. These *in situ* injectable PRP-laden GM hydrogels (GM@PRP) induced the proliferation, migration, and differentiation of rBMSCs as well as promoted macrophages polarization from M1 to M2 phenotypes *in vitro* [41]. Moreover, these hydrogels promoted the regeneration of tibial defects [7, 23]. A battery of *in vitro* assays, such as scratch wound healing assay, Transwell migration assay, and tubule-like network formation assay exhibited remarkable wound healing, cell migration, and angiogenic networks formation. We surmise that the PRP-mediated GFs may induce above-mentioned therapeutic effects in GM@PRP hydrogels, which may also have implications to simultaneously promote osteogenesis and angiogenesis for bone tissue repair *in vivo* [12, 55, 56].

We separately prepared GM and PRP solutions and fabricated a range of hydrogels by varying the content of the PRP. The GM_x_@PRP (*x *=* *1, 2, 3, and 4) were prepared to screen for the effect of PRP on the mechanical properties of hydrogels (Supplementary Table S3). The GM@PRP1 hydrogels, which were incorporated with an equal ratio of GM and PRP, displayed a delicate balance between mechanical properties and microstructure of the hydrogels (Figure 2A). Further increase of the PRP not only destabilized the structure of the hydrogels but also adversely impacted their mechanical properties. It is worthy to note that the higher content of the GM may lead to a dense structure, which may not be conducive for cell growth and infiltration. Intriguingly, photo-crosslinked GM@PRP scaffolds afforded sustained release of bioactive cues while maintaining structural integrity and avoiding fast degradation *in vitro* (Figure 2).

Whole transcriptome RNA sequencing analysis further established the pivotal role of the PRP to regulate cellular behaviors, which promoted the expression of multiple signaling pathways in both rBMSCs and HUVECs. Metabolism, vascular, and biomineralization related pathways were activated, which in turn improved the metabolic function, cell proliferation, and biomineralization. Equally important, the PRP promoted angiogenesis and cell migration related signaling pathways, while suppressed inflammatory and cell apoptosis associated signaling pathways (Figures 5 and 6). Our results are also in agreement with the previous findings about the PRP. It has been previously shown that the PRP confers a reparative environment by promoting the macrophages polarization towards M2 phenotypes alongside stimulating osteochondral regeneration [7]. Altogether, these data indicated that the PRP may be instrumental to promote cell metabolism/viability as well as increase vascularization and immuno-modulation in GM@PRP hydrogels to govern tissue repair.

In addition to the regulation of multiple cellular processes *in vitro,* GM@PRP hydrogels also promoted the regeneration of the segmental tibia defects (those smaller than that of 2 mm) albeit their poor performance for the repair of large-sized defects (4 mm). The smaller tibia defects showed *de novo* bone formation alongside mild bone hyperplasia, thereby showing good potential of GM@PRP hydrogels for osteochondral tissue repair. *De novo* bone tissues manifested seamless interfacial integration as well as good connective tissue repair [10]. Meanwhile, *in vivo* results showed higher collagen production, neovascularization, and greater osteogenic activity. These improvements may be ascribed to the PRP and GM@PRP hydrogels; the latter are reminiscent of bone microenvironment. The PRP has multifunctional benefits to confer an angiogenic microenvironment through the spatiotemporal release of cytokines and chemokines as well as an improved cell homing and possibly the differentiation of the recruited cells into the targeted phenotypes. Interestingly, the scaffolds showed a significant reparative effect for bone tissue repair; the regeneration efficiency can be further improved by increasing the pore size of the hydrogel, incorporation of functional cells, and modification of the application form of hydrogels using various fabrication methods, such as 3D printing. Further optimizations are required to improve rate of tissue infiltration and avoid bone hyperplasia to promote large-sized bone defect regeneration. To put together, GM@PRP hydrogels could simultaneously induce angiogenesis and osteogenesis for segmental bone defect regeneration.

Notwithstanding, there are also several limitations of this study which warrant future studies. We have not deciphered the detailed mechanism of the GF-binding affinity of the PRP; the latter may have varying binding affiniti for different types of GFs. Nevertheless, further in-depth studies are needed to decipher the GFs binding affinity of these PRP-laden hydrogels. We observed poor performance of GM@PRP hydrogels for the regeneration of large-sized bone defects, plausibly due to bone hyperplasia, which may compromise vascularization and biomineralization. Moreover, we observed limited cell infiltration into the implanted hydrogels *in* vivo, which may also limit the healing of the bone defects. The directional regeneration of large-sized segmental bones is also important, which requires further optimization of implants to better promote vascularization and osteogenic differentiation. For *in vivo* evaluation in a tibial defect model, we only transplanted GM@PRP hydrogels and did not transplant GM hydrogel. Therefore, it is necessary to implant GM hydrogels to closely clarify the regeneration mechanism of the GM@PRP hydrogels. It is worthy to note that the GM@PRP hydrogels cannot meet the stringent requirements of mechanical properties for osteochondral tissue repair. These *in situ* injectable hydrogels may also have potential prospects for the other non-load bearing applications, such as infarcted myocardium repair, skeletal muscle defect repair, and so on. Nevertheless, further optimizations are required to improve rate of tissue infiltration and avoid bone hyperplasia to promote large-sized bone defect regeneration. Therefore, alternative approaches are required to further strengthen the mechanical properties of hydrogels to broaden their application for the other osteochondral defects, such as the utilization of appropriate cross-linking agents or the incorporation of reinforcing agents or supporting polymers into these hydrogels [57]. Moreover, the further optimizations are required to improve rate of tissue infiltration and avoid bone hyperplasia to promote large-sized bone defect regeneration.

## Conclusions

We developed *in situ* injectable hydrogels loaded with autologous PRP through the physical blending of the GM and PRP (GM@PRP) in appropriate ratios and exploited them for the regeneration of large-sized tibial defects in rats. The GM@PRP hydrogels exhibited an excellent injectability, sustained release of different types of GFs, and degradability alongside good cytocompatibility, angiogenesis, and chemotaxis *in vitro*. The GM@PRP hydrogels may promote bone defect healing through several processes, including: (i) Migration of HUVECs and rBMSCs, (ii) formation of angiogenic networks, (iii) collagen deposition, (iv) immuno-modulation, (v) release of bioactive molecules, and (vi) simultaneous vascularization and osteogenesis. Taken together, these *in situ* injectable GM@PRP hydrogels may have broad implications for the regeneration of bone defects and may also be worthy for the other related disciplines.

## Supplementary Material

rbae022_Supplementary_Data
