# Supplementary material for: Methacrylated gelatin and platelet-rich plasma based hydrogels promote regeneration of critical-sized bone defects
Source: Regen Biomater. 2024 Mar 5;11:rbae022. doi: 10.1093/rb/rbae022 (PMC10985677; doi:10.1093/rb/rbae022)
Supplement: rbae022_Supplementary_Data [file rbae022_supplementary_data.zip › Revised_Supporting_information.docx]

**Methacrylated Gelatin and Platelet-Rich Plasma Based Hydrogels Promote Regeneration of Critical-Sized Bone Defects**

Shichao Lian ^a,b^, Zhiyu Mu ^c^, Zhengchao Yuan ^d, *^, Muhammad Shafiq^e^, Xiumei Mo ^d, *^, Weidong Mu ^a, *^.

^a^ Department of Traumatic Orthopaedics, Shandong Provincial Hospital, Shandong University, Jinan, Shandong, 250012, China.

^b^ Zoucheng People's Hospital, Zoucheng, Shandong, 273500, China.

^c^ Medical Physics and Biomedical Engineering, University of London, Gower Street, London, WC1E 6BT, England.

^d^ State Key Laboratory for Modification of Chemical Fibers and Polymer Materials, Shanghai Engineering Research Center of Nano-Biomaterials and Regenerative Medicine, College of Biological Science and Medical Engineering, Donghua University, Shanghai, 201620, PR China.

^e^ Innovation Center of NanoMedicine (iCONM), Kawasaki Institute of Industrial Promotion, Kawasaki-ku, Kawasaki 210-0821, Japan.

^*^Corresponding authors. Zhengchao Yuan (yuanzhengchao2021@163.com), Xiumei Mo (xmm@dhu.edu.cn), and Weidong Mu (sdslmwd@126.com).

**List of Supplementary Tables**

Table S1: Composition of the different types of hydrogels along with the varying ratios of PRP and GM.

Table S2: Identification codes of samples used for the whole transcriptome RNA sequencing.

Table S3: Summary of the hydrogels used for transplantation into a tibial defect model *in vivo* along with the dimensions of the defects.

Table S4: Dimensions of the tibial defects at different time points after the implantation of hydrogels 6 weeks post-operatively in SD rats.

**List of Supplementary Figures**

Fig. S1 Fig. S1 Schematic illustration of the preparation of platelet rich plasma (PRP).

Fig. S2 Standard curves for the release kinetics of different types of growth factors, such as EGF, PDGF, and TGF-β.

Fig.S3 Schematic diagram of tibia and tibia size measurement. (a) the Key points of tibial defect model, (b) length measurement of tibia, (c) diameter measurement of different tibia position of 6-weeks’ SD rat. (d) Different tibia for measurement of 6-weeks’ SD rat.

Fig. S4 (a) Preparation of segmental tibial defects in rats. (b) Digital pictures of tibial defects of 1, 2, and 4 mm.

Fig. S5 Representative images of GM hydrogels adhered to (a) bone surface and (b) bone defection.

Fig. S6 (a) Representative time-stress curves of the Lap-shear test. (b) Lap-shear strength of GM and GM@PRP hydrogels (n =5).

Fig. S7 Degradation ratio of GM and GM@PRP hydrogels

Fig. S8 Flow cytometry assay of CD29, CD90, CD44, CD45, CD34, CD11b, IgG, IgG1, IgG2a by using rBMSCs.

Fig. S9 (a-b) The digital pictures and (c-d) the CT pictures of normal tibial.

Fig. S10 H&E staining (a) and MT staining (b) of repaired bone regeneration at 4 weeks. Scale bars, 2 mm, 600 μm and 200 μm.

Fig. S11 H&E staining and MT staining of normal tibial bones. Scale bars, 2 mm, 600 μm and 200 μm.

Fig. S12 Immunofluorescence staining of Col-I of normal tibial bone. Scale bars, 2 mm, 800 μm and 300 μm.

Fig. S13 Immunofluorescence staining for α-SMA and CD31 (a) and OPN/OCN (b) of normal tibial bone. Scale bars, 2 mm, 800 μm and 300 μm.

Table S1: Composition of the different types of hydrogels along with the varying ratios of PRP and GM.

| Name | GM（10wt%） | PRP (100%) | Ratio (v:v) |
| --- | --- | --- | --- |
| GM | 1 mL | None | —— |
| GM@PRP | 1 mL | 1 mL | 1:1 |
| GM2@PRP | 2 mL | 1 mL | 2:1 |
| GM3@PRP | 3 mL | 1 mL | 3:1 |
| GM4@PRP | 4 mL | 1 mL | 4:1 |

Table S2: Identification codes of samples used for the whole transcriptome RNA sequencing.

| Name | Sample | Control groups | PRP groups |
| --- | --- | --- | --- |
|  |  | Treating without PRP | Treating PRP |
| BMSCs | 1 | CB-1 | PB-1 |
|  | 2 | CB-2 | PB-2 |
|  | 3 | CB-3 | PB-3 |
| HUVECs | 1 | CH-1 | PH-1 |
|  | 2 | CH-2 | PH-2 |
|  | 3 | CH-3 | PH-3 |

Table S3: Summary of the hydrogels used for transplantation into a tibial defect model *in vivo* along with the dimensions of the defects.

| Name | Defection length | Filling hydrogel | Hydrogel (Filling length) |
| --- | --- | --- | --- |
| Control | 1 mm | None | None |
| GM@PRP-1 | 1 mm | GM@PRP | 1 mm |
| GM@PRP-2 | 2 mm | GM@PRP | 2 mm |
| GM@PRP-4 | 4 mm | GM@PRP | 4 mm |

Table S4: Dimensions of the tibial defects at different time points after the implantation of hydrogels 6 weeks post-operatively in SD rats.

| Name | Length | Diameter | Diameter | Diameter | Diameter |
| --- | --- | --- | --- | --- | --- |
|  | (cm) | D1(mm) | D2 (mm) | D3 (mm) | D4 (mm) |
| Minimum diameter | 3.64±0.11 | 2.35±0.46 | 2.01±0.13 | 2.73±0.23 | 3.92±0.55 |
| Maximum diameter |  | 2.99±0.24 | 2.68±0.31 | 3.56±0.44 | 4.64±0.54 |
| Average diameter |  | 2.67±0.48 | 2.34±0.41 | 3.15±0.55 | 4.28±0.65 |


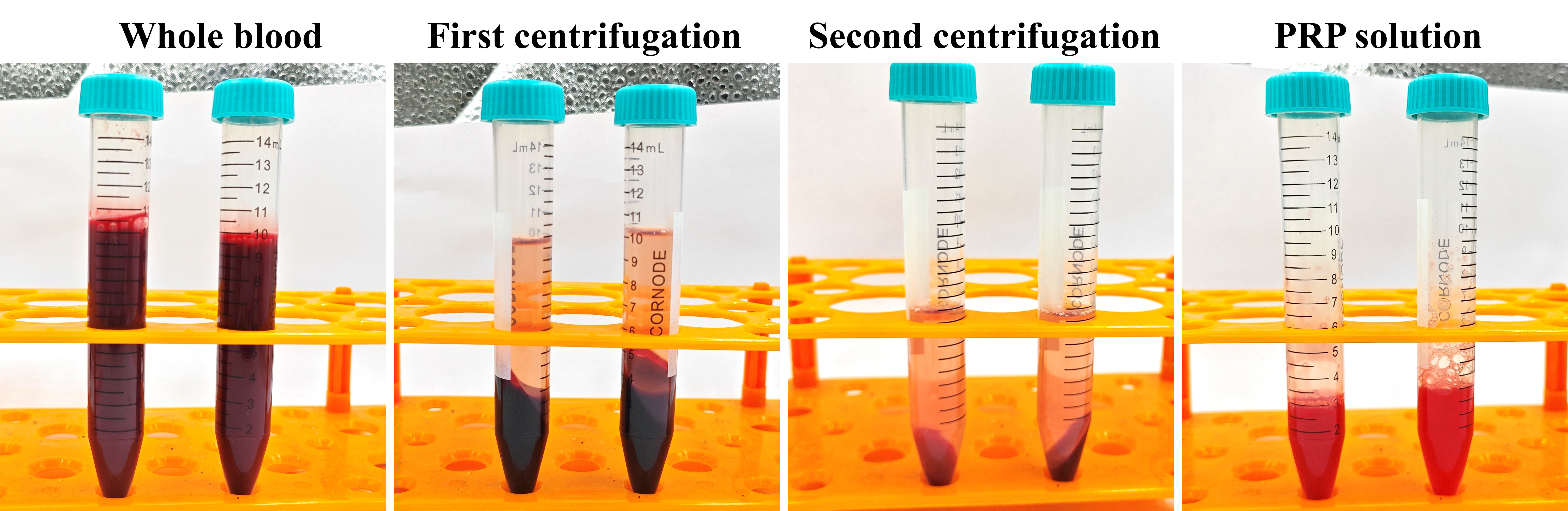


Fig. S1 Schematic illustration of the preparation of platelet rich plasma (PRP).


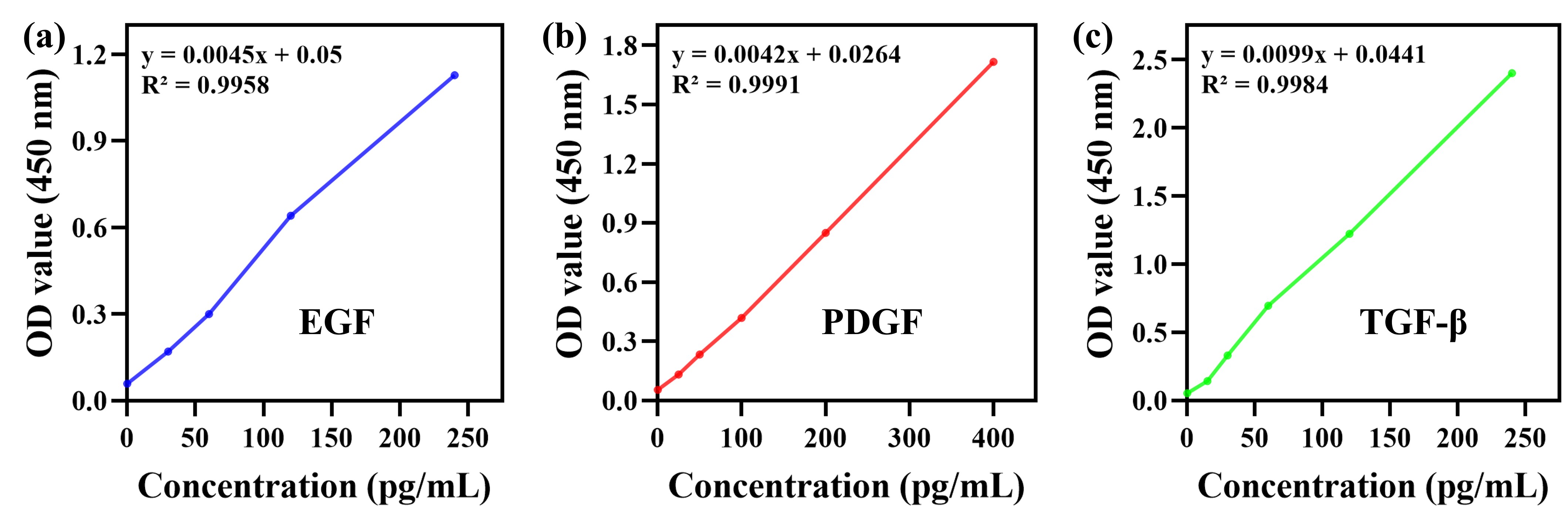


Fig. S2 Standard curves for the release kinetics of different types of growth factors, such as EGF, PDGF, and TGF-β.


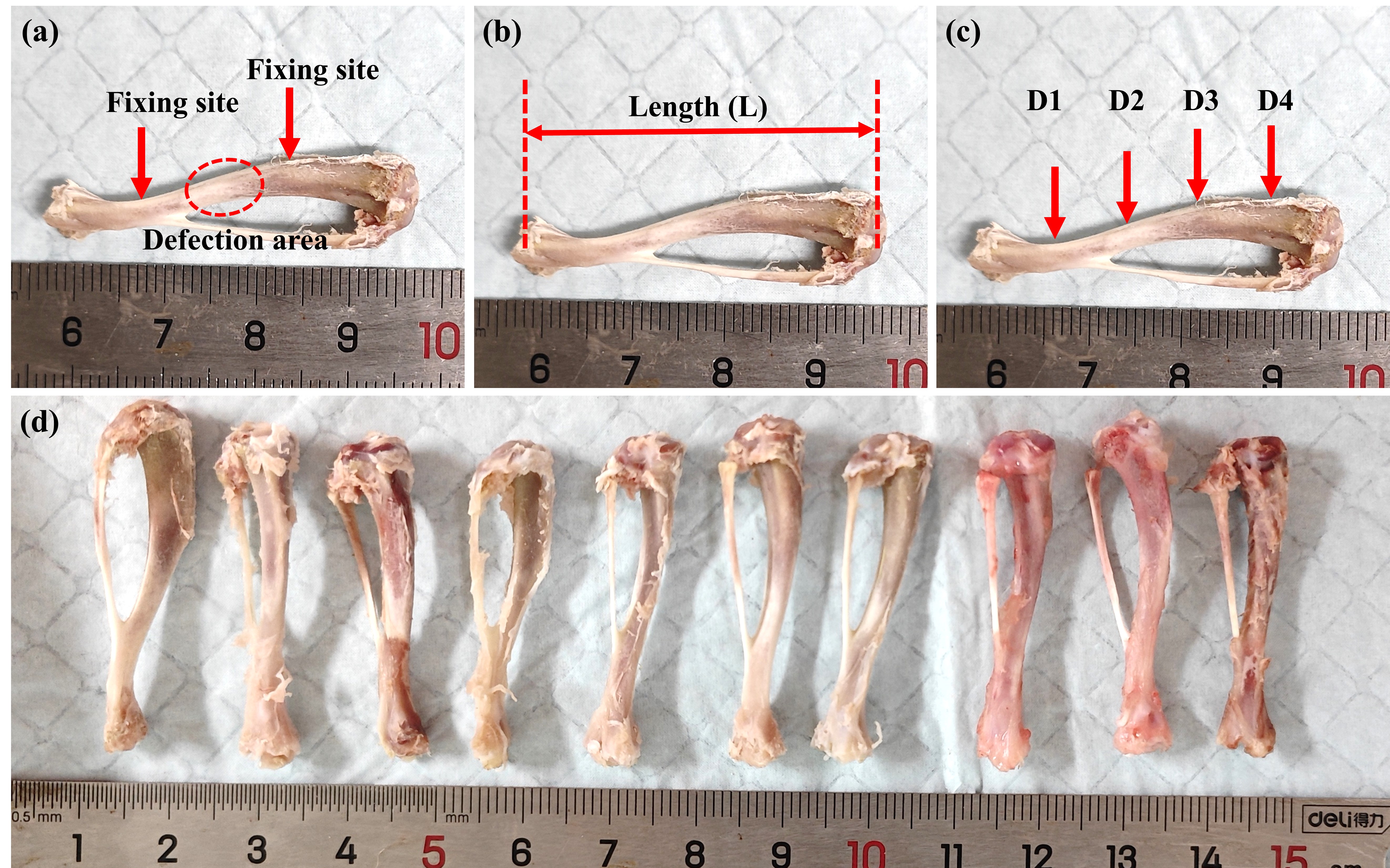


Fig.S3 Schematic diagram of tibia and tibia size measurement. (a) the Key points of tibial defect model, (b) length measurement of tibia, (c) diameter measurement of different tibia position of 6-weeks’ SD rat. (d) Different tibia for measurement of 6-weeks’ SD rat.


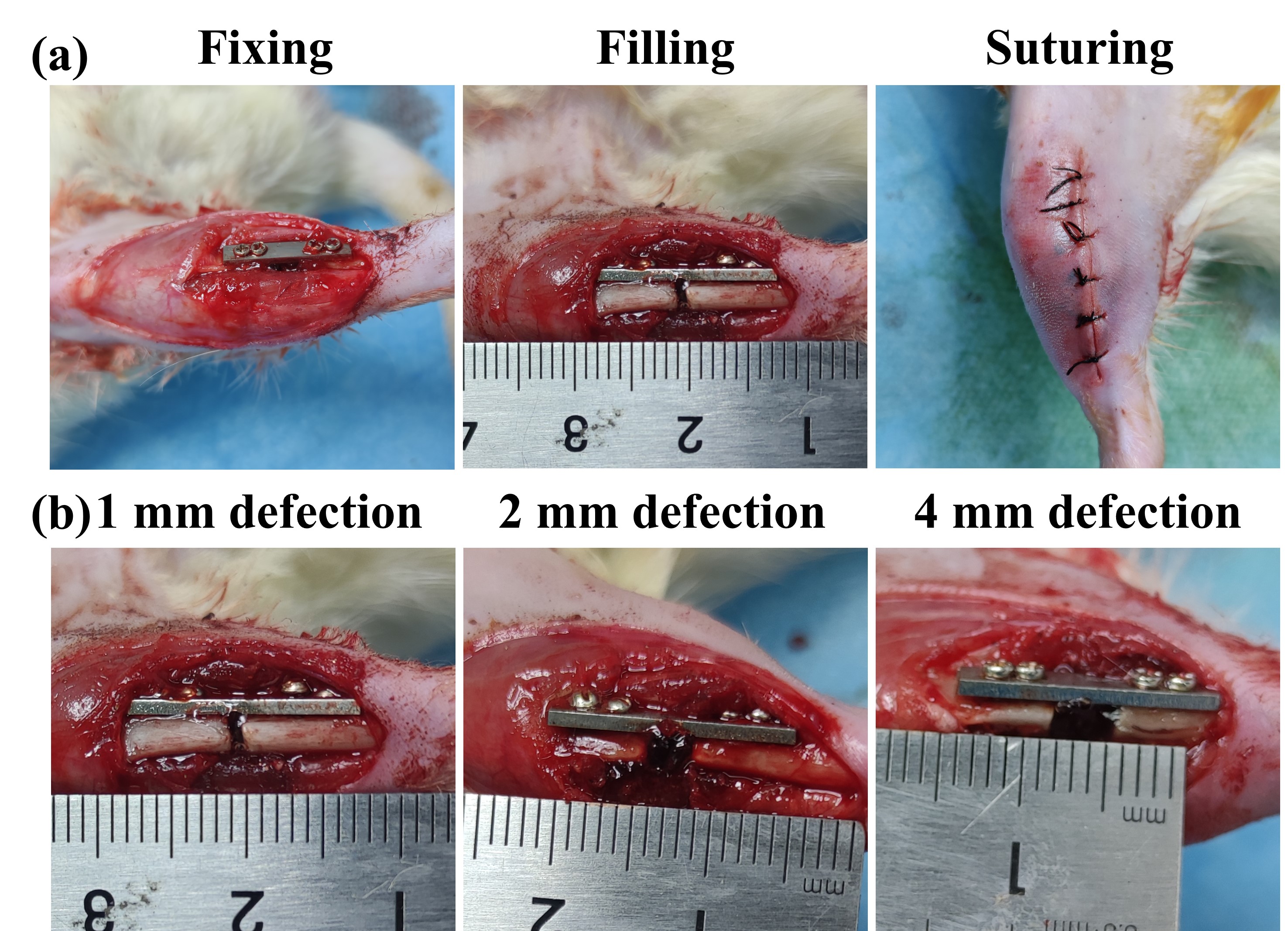


Fig. S4 (a) Preparation of segmental tibial defects in rats. (b) Digital pictures of tibial defects of 1, 2, and 4 mm.


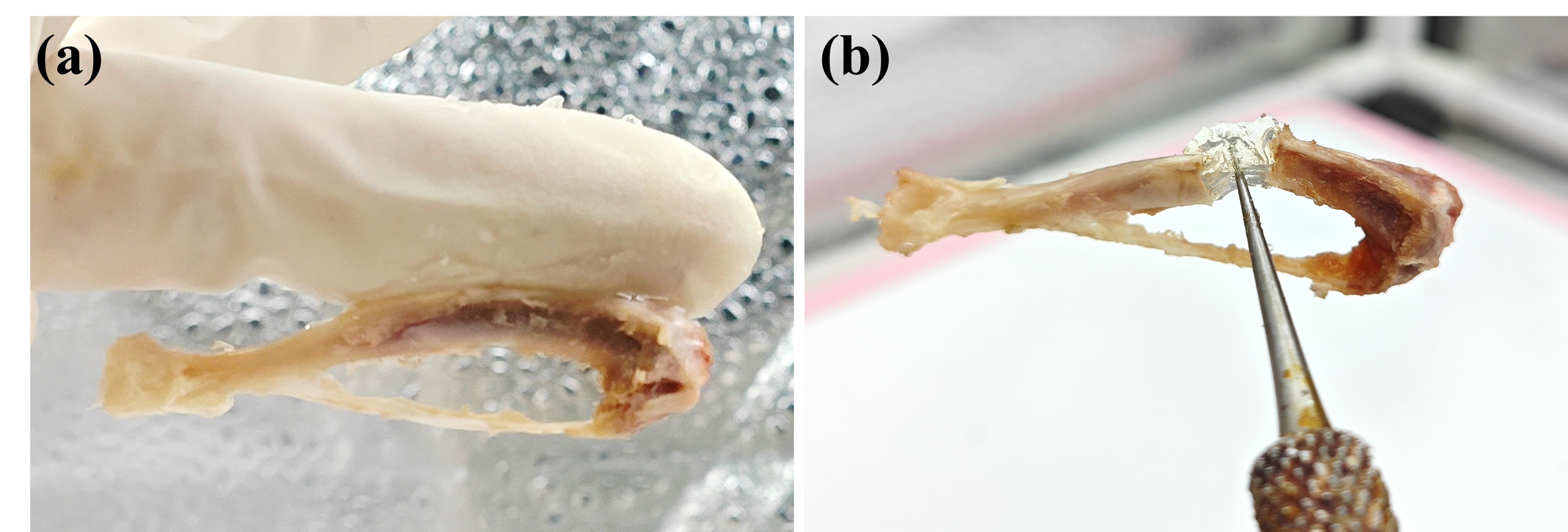


Fig. S5 Representative images of GM hydrogels adhered to (a) bone surface and (b) bone defection.


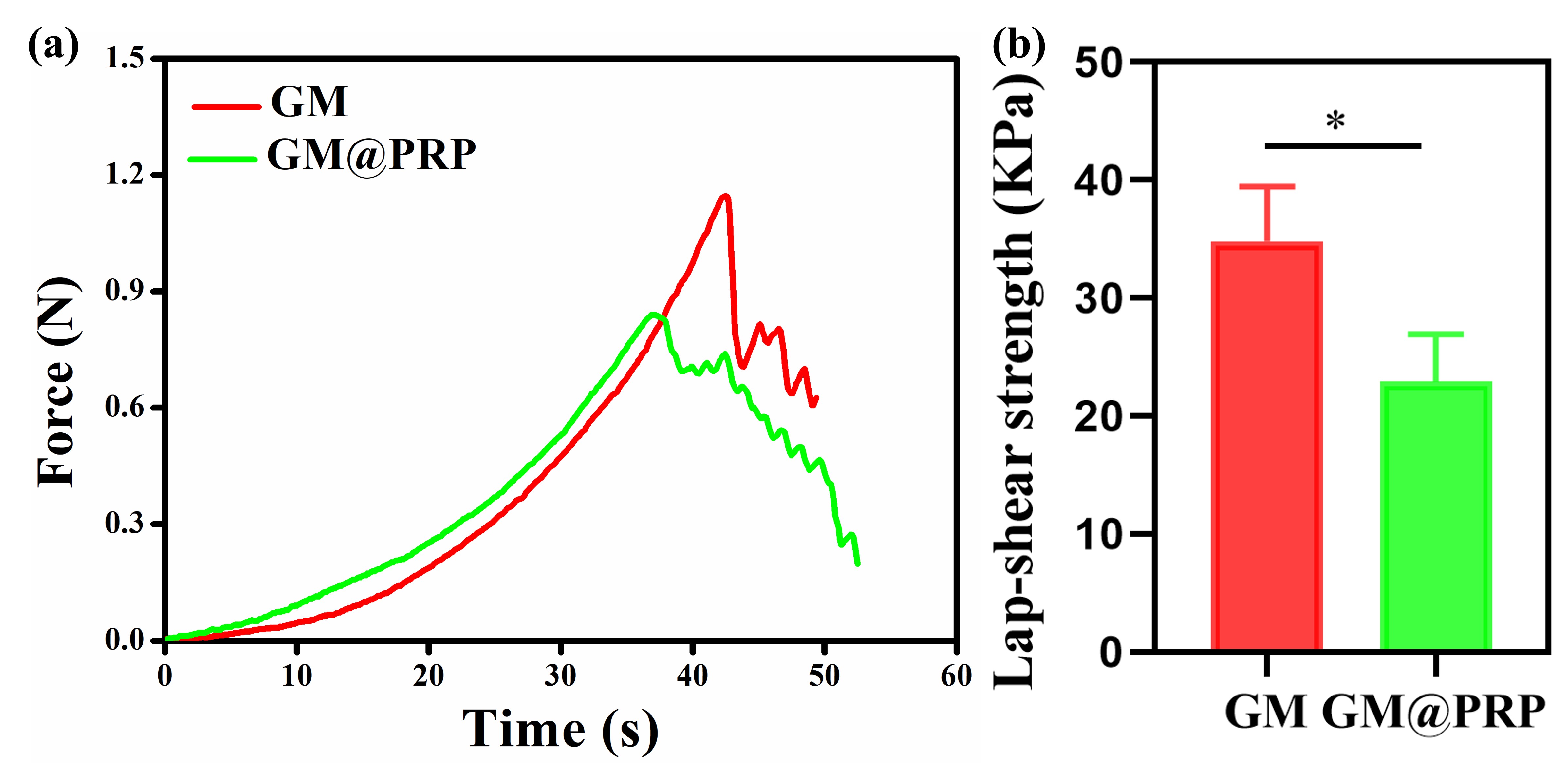


Fig. S6 (a) Representative time-stress curves in a Lap-shear test. (b) Lap-shear strength of GM and GM@PRP hydrogels (n =5).


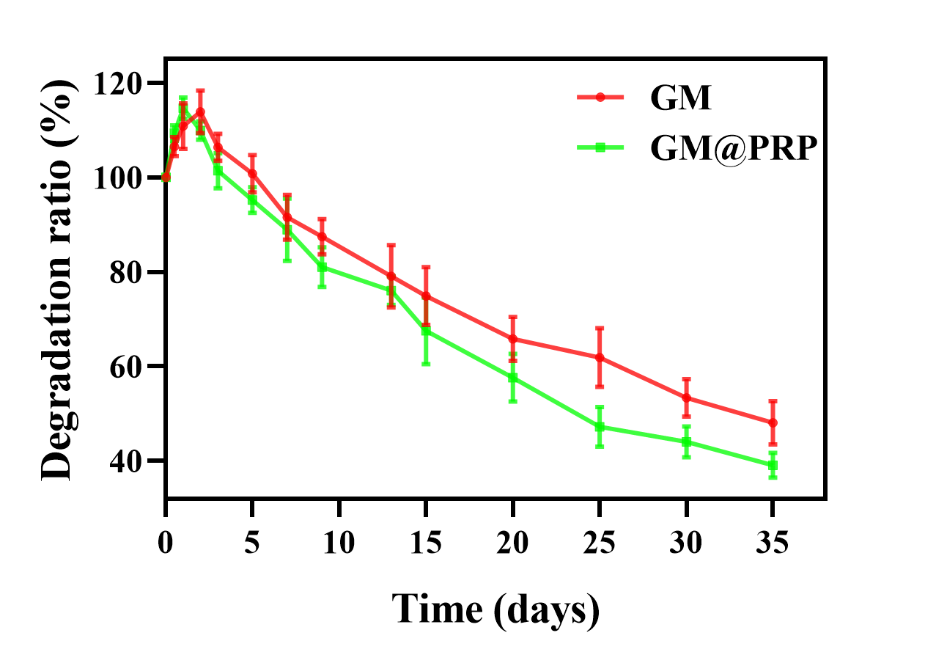


Fig. S7 The degradation ratio of GM and GM@PRP hydrogels.


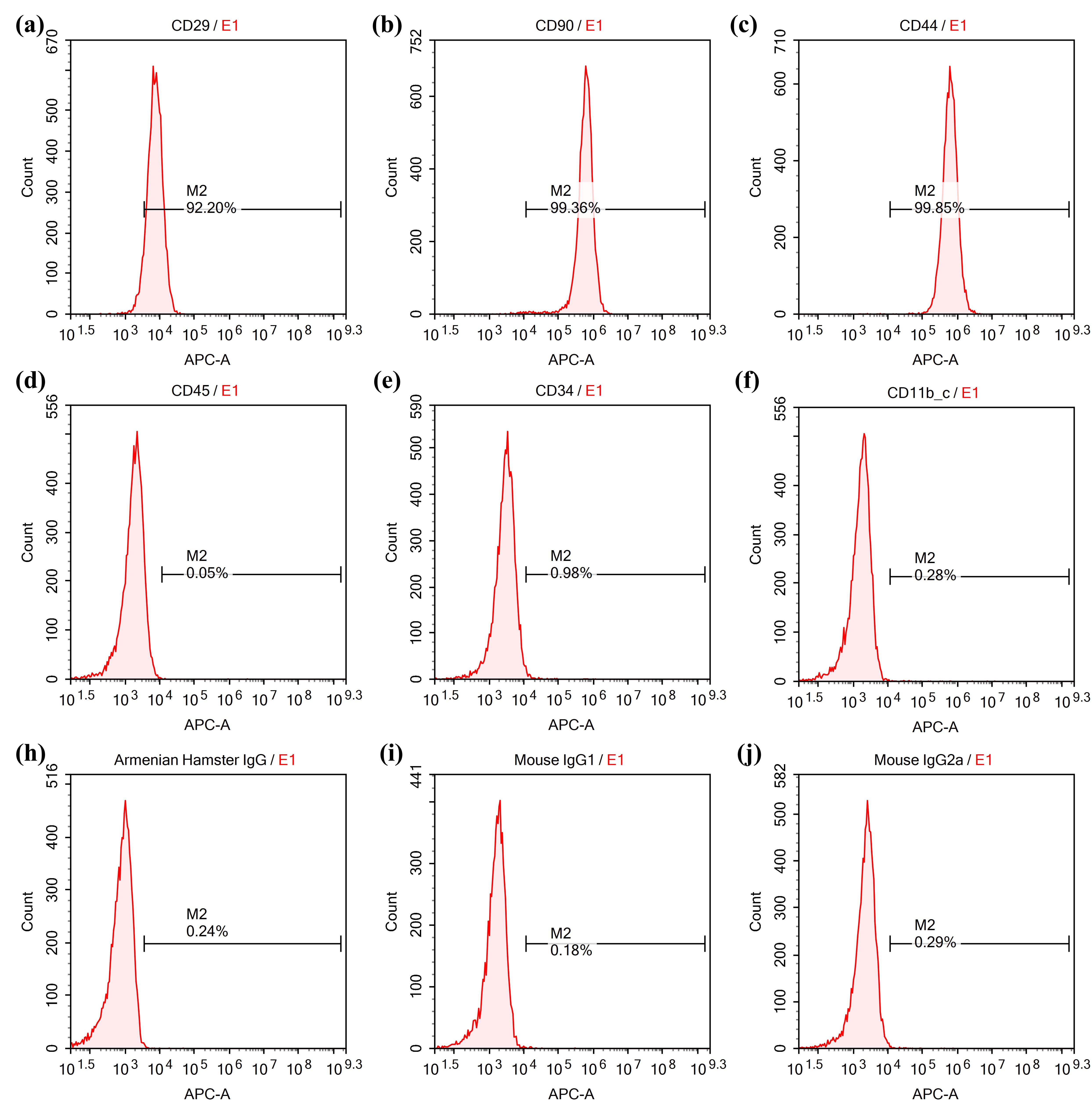


Fig. S8 Flow cytometry assay of CD29, CD90, CD44, CD45, CD34, CD11b, IgG, IgG1, IgG2a by using rBMSCs.


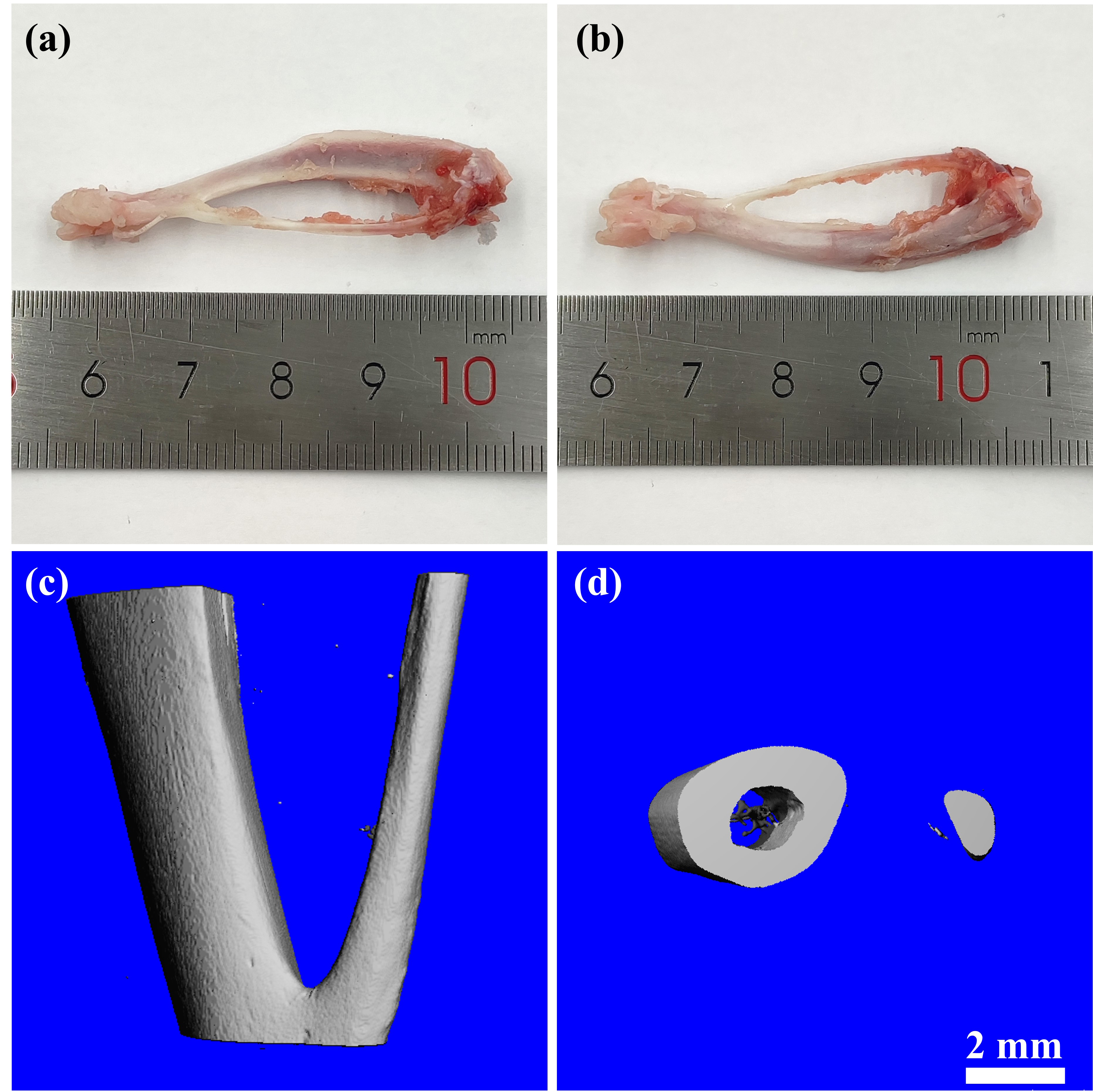


Fig. S9 (a-b) Digital pictures and (c-d) the CT pictures of normal tibial.


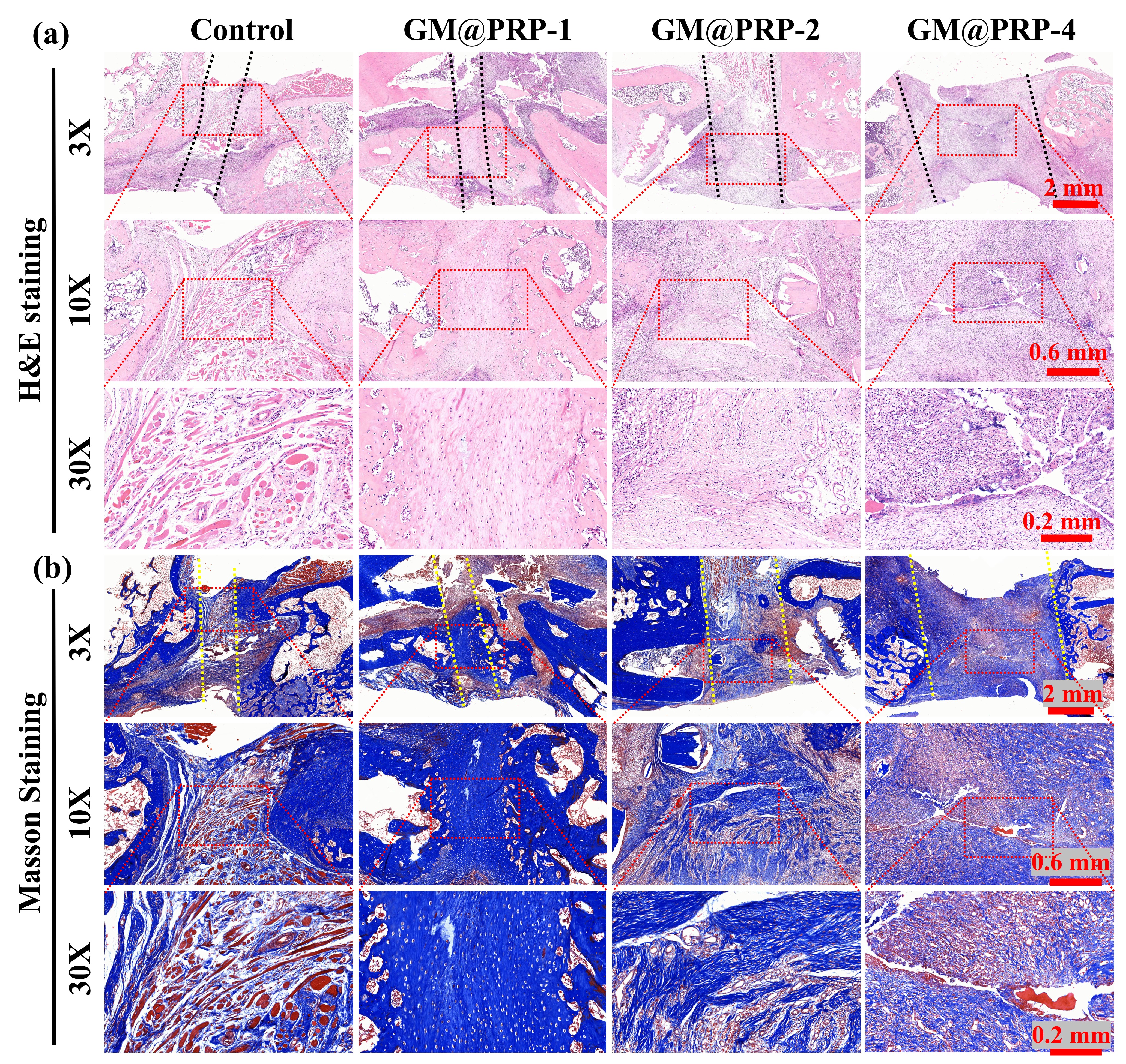


Fig. S10 H&E staining (a) and MT staining (b) of repaired bone regeneration at 4 weeks. Scale bars, 2 mm, 600 μm and 200 μm.


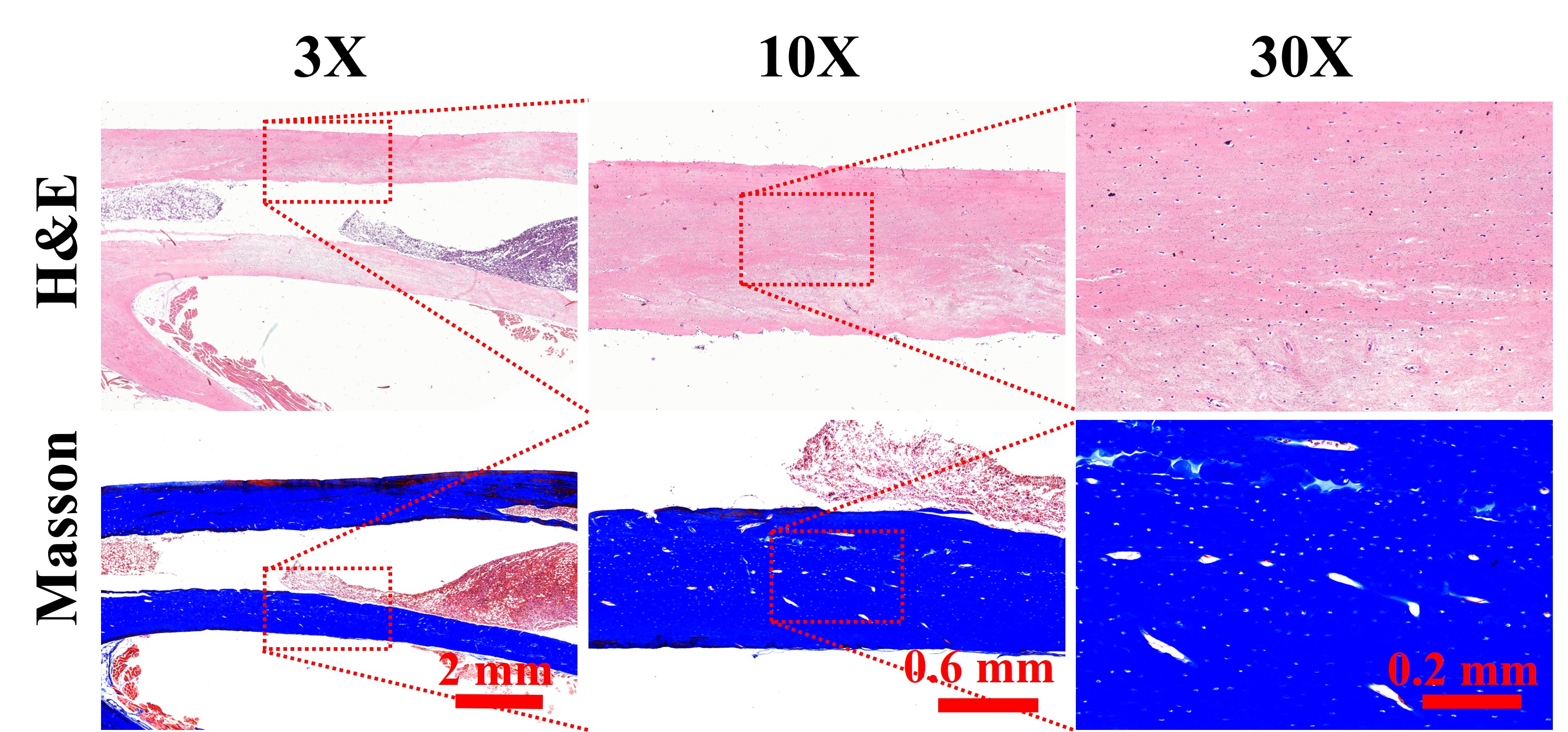


Fig. S11 H&E staining and MT staining of normal tibial bones. Scale bars, 2 mm, 600 μm and 200 μm.


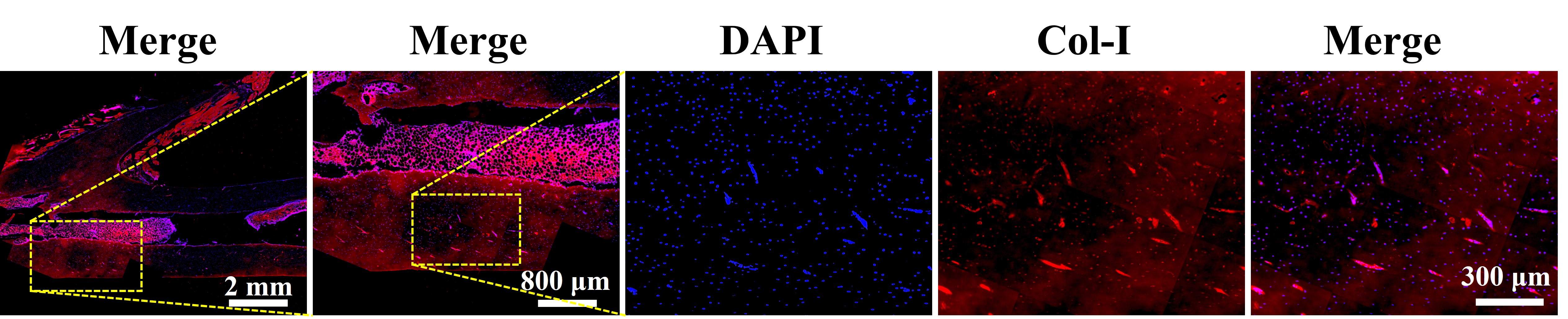


Fig. S12 Immunofluorescence staining of Col-I of normal tibial bone. Scale bars, 2 mm, 800 μm and 300 μm.


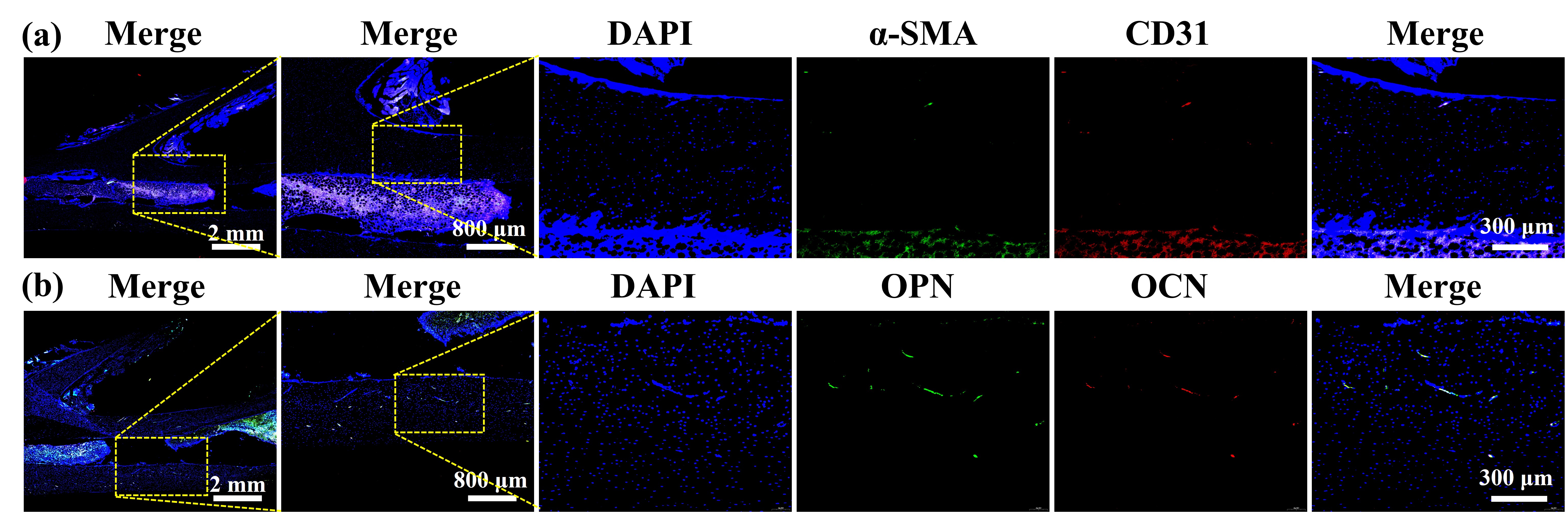


Fig. S13 Immunofluorescence staining for α-SMA and CD31 (a) and OPN/OCN (b) of normal tibial bone. Scale bars, 2 mm, 800 μm and 300 μm.
