# Supplementary material for: Methacrylated gelatin and platelet-rich plasma based hydrogels promote regeneration of critical-sized bone defects
Source: Regen Biomater. 2024 Mar 5;11:rbae022. doi: 10.1093/rb/rbae022 (PMC10985677; doi:10.1093/rb/rbae022)
Supplement: rbae022_Supplementary_Data [file rbae022_supplementary_data.zip › Response to Editorial Comments.docx]

**Response to Editorial Comments**

We are very grateful to the editorial office about the comment of our accepted manuscript (Ref. # RB-2023-369.R1) entitled *“Methacrylated Gelatin and Platelet-Rich Plasma Based Hydrogels Promote Regeneration of Critical-Sized Bone Defects”*. In the light of the editorial comments, we have clearly stated that the animal study was approved by the ethical committee and have also included the ethical approval number for this animal study. The detailed response to the editorial comment is as below, please:

**Comment**: Please state it if the animal study was approved by the ethical committee and the ethical approval number for this animal study must be included in the main text.

**Reply:** We have clearly stated that the animal study was approved by the ethical and have also included the ethical approval number for this animal study.

Please see section # 2.5 titled “Animal Experiments”

**2.5 Animal experiments**

The animal study was approved by the Experimental Animal Ethics Committee of Shandong Provincial Hospital and the ethical approval number for this animal study is No. 2022-115.

We are once again very grateful to the editorial board and look forward to contribute further to Regenerative Biomaterials.
